# Sortilin restricts secretion of apolipoprotein B-100 by hepatocytes under stressed but not basal conditions

**DOI:** 10.1172/JCI144334

**Published:** 2022-03-15

**Authors:** Donna M. Conlon, Carolin V. Schneider, Yi-An Ko, Amrith Rodrigues, Kathy Guo, Nicholas J. Hand, Daniel J. Rader

**Affiliations:** 1 Division of Translational Medicine and Human Genetics, Department of Medicine and; 2 Department of Genetics, Perelman School of Medicine at the University of Pennsylvania, Philadelphia, Pennsylvania, USA.

**Keywords:** Genetics, Vascular Biology, Lipoproteins

## Abstract

Genetic variants at the *SORT1* locus in humans, which cause increased *SORT1* expression in the liver, are significantly associated with reduced plasma levels of LDL cholesterol and apolipoprotein B (apoB). However, the role of hepatic sortilin remains controversial, as genetic deletion of sortilin in mice has resulted in variable and conflicting effects on apoB secretion. Here, we found that *Sort1*-KO mice on a chow diet and several *Sort1*-deficient hepatocyte lines displayed no difference in apoB secretion. When these models were challenged with high-fat diet or ER stress, the loss of *Sort1* expression resulted in a significant increase in apoB-100 secretion. *Sort1*-overexpression studies yielded reciprocal results. Importantly, carriers of *SORT1* variant with diabetes had larger decreases in plasma apoB, TG, and VLDL and LDL particle number as compared with people without diabetes with the same variants. We conclude that, under basal nonstressed conditions, loss of sortilin has little effect on hepatocyte apoB secretion, whereas, in the setting of lipid loading or ER stress, sortilin deficiency leads to increased apoB secretion. These results are consistent with the directionality of effect in human genetics studies and suggest that, under stress conditions, hepatic sortilin directs apoB toward lysosomal degradation rather than secretion, potentially serving as a quality control step in the apoB secretion pathway in hepatocytes.

## Graphical abstract

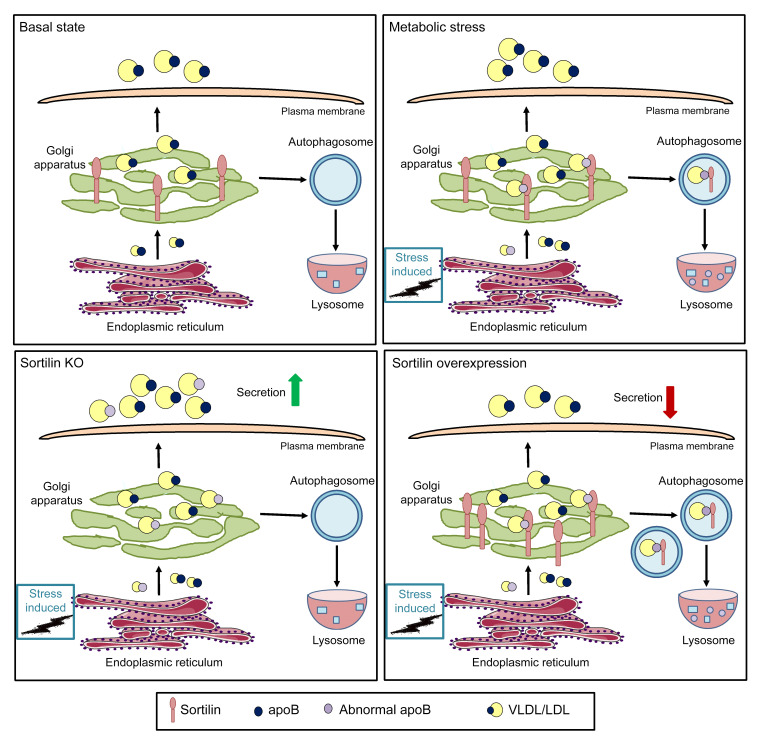

## Introduction

The protein sortilin, encoded by the gene *SORT1*, has been of great interest to the genetics and cardiometabolic disease communities over the last decade. Sortilin is a type I transmembrane multiligand receptor that is synthesized as a propeptide in the endoplasmic reticulum (ER) and processed to its mature form in the Golgi. It is best known for its role in trafficking proteins from the Golgi to endolysosomal compartments, where it deposits cargo targeted for degradation in the lysosome, and then is trafficked back to the Golgi via the retromer complex (1–4). The initial discovery that noncoding genetic variants at a locus on chromosome 1p13.3 near *SORT1* were highly significantly associated with LDL cholesterol (LDL-C; ref. 5) and coronary artery disease (CAD; ref. 6) was remarkable, because only previously known regulators of LDL-C metabolism, such as *LDLR*, *APOB*, and *PCSK9*, had comparably significant associations of common variants with LDL-C (7). Most recently, in more than 300,000 people (8) the lead *SORT1* variant rs12740374 was associated with a 6 mg/dL reduction in LDL-C (*P =* 1 × 10^–323^) and a 11% reduction in risk of CAD (*P =* 1 × 10^–23^). This same variant was also associated with a significant decrease in plasma apolipoprotein B (apoB) levels (*P <* 1 × 10^–300^) in White participants in the UK Biobank (9) as well as in people of other ancestries (10). This LDL-C– and apoB-lowering variant, which creates a new binding site for CCAAT/enhancer-binding protein α, is associated with 12-fold increased expression of *SORT1* specifically in the liver (11), suggesting that increased hepatic expression of *SORT1* reduces LDL-C levels. Indeed, we (11, 12) and others (13–15) have reported that hepatic overexpression of *SORT1* in mice reduces LDL-C, at least in part, through reduction in the hepatic production of its precursor VLDLs. However, the reported effects of the genetic loss of sortilin on VLDL and apoB-100 secretion have been contradictory and have led to confusion in the field. While initial reports of global *Sort1*-KO mouse models suggested that VLDL production was reduced (12, 16), other reports have suggested that loss of sortilin led to increased VLDL production (13, 17). Thus, the physiological role of sortilin in hepatocyte VLDL and apoB-100 secretion remains unclear (18).

The assembly and secretion of VLDL by hepatocytes is a highly complex process that has been extensively studied (19, 20). ApoB-100 is the primary and essential structural protein in VLDL, and it is constitutively synthesized by hepatocytes, generating an intracellular pool of this large hydrophobic protein that must be both co- and posttranslationally loaded with lipid in order for it to assume the proper conformation in the ER and to enter the secretory pathway (21). The regulation of apoB-100 during its translation and its associated degradation in the ER (ER-associated degradation [ERAD]) is critical and has been extensively studied (19, 21). Importantly, its regulation in post-ER compartments, specifically the Golgi, has also received attention in recent years. Post-ER presecretory proteolysis (PERPP) refers to the regulated degradation of apoB-100 after it exits the ER, a process that has been proven to be an important element of the apoB-100 production pathway (22). PERPP results primarily in the lysosomal degradation of apoB-100 as opposed to the proteosomal degradation of apoB-100 during ERAD. Important examples of PERPP degradation of apoB-100 in the Golgi include the uptake of apoB aggregates caused by polyunsaturated fatty acid treatment or increased insulin signaling by autophagosomes that traffic their cargo to the lysosome for degradation (20, 23–27). Several studies have implicated sortilin interaction with apoB-100 in the Golgi as potential regulator of apoB-100 secretion (12, 15, 25), but understanding the directionality by which hepatic sortilin affects apoB-100 secretion is of critical importance to the field (28).

Here, we report detailed tracer studies of apoB-100 secretion in mice lacking sortilin, globally or specifically in hepatocytes, as well as in cultured hepatocyte cell lines lacking sortilin. We found that, under basal nonstressed conditions, neither deletion nor overexpression of hepatocyte sortilin had discernible effects on apoB-100 secretion. However, under conditions that increase apoB secretion or induce ER stress, deletion of hepatocyte sortilin led to a clear increase in apoB-100 secretion, whereas overexpression led to a significant decrease in apoB-100 secretion. We also found that the LDL-reducing variant at the *SORT1* locus has a significantly greater effect in people with diabetes compared with those without diabetes. These data are consistent with the directionality of the human genetic data and suggest a unifying hypothesis regarding the physiological role of hepatic sortilin in reducing apoB-100 secretion under conditions of metabolic or ER stress.

## Results

### Global Sort1 and hepatocyte-specific Sort1-deleted mice have no difference in apoB-100 secretion compared with WT mice under basal conditions.

**Figure 1:**
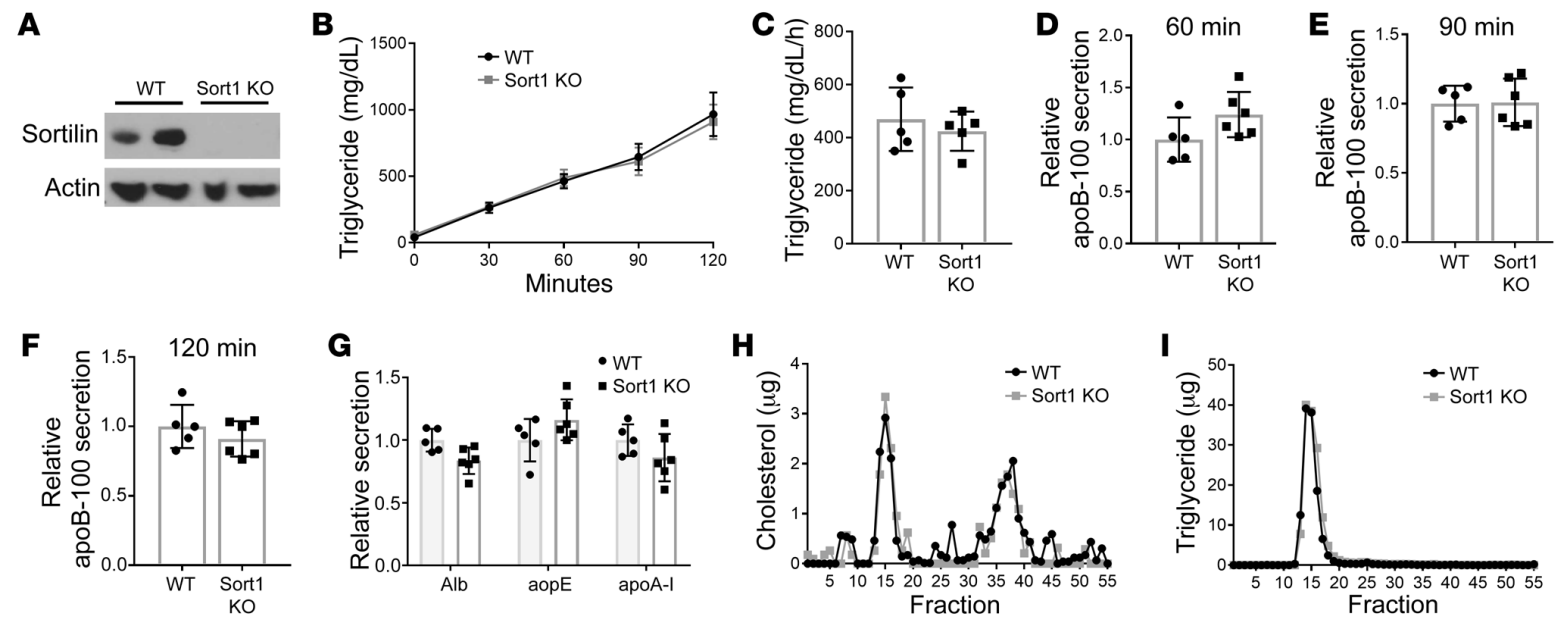
Global Sort1-deleted mice under basal conditions have no difference in apoB or TG secretion compared with WT mice. 8- to 10-week old WT and *Sort1*-KO mice were used for experiments. (**A**) Liver homogenate was Western blotted for sortilin and actin protein. (**B**) Mice were injected i.v. with Pluronic and ^35^S Met/Cys and bled every 30 minutes for 2 hours. Plasma TG was measured. (**C**) TG secretion rate was calculated as the mg/dL/h. (**D**–**F**) Total plasma from each mouse at 60, 90, and 120 minutes after injection was subjected to autoradiography, and ^35^S-labeled apoB-100 was quantified by liquid scintillation counting. (**G**) ^35^S-labeled albumin, apoE, and apoA-I bands were also counted in 120-minute total plasma. *n =* 5/group. Representative of 4 independent experiments. (**H** and **I**) In 1 experiment, 2-hour plasma was pooled within each group and separated by FPLC. (**H**) Total cholesterol and (**I**) TG were measured in each fraction.

We studied global *Sort1*-KO mice on a C57BL6/J background (29) as compared with their WT littermate controls; both groups of mice were maintained on a chow diet. As expected, there was no detectable sortilin protein in the livers of the KO mice (Figure 1A). There was no difference between genotypes in body weight, plasma cholesterol, or plasma triglyceride (TG) in mice in WT versus Sort1-KO mice maintained on a chow diet (Table 1). Mice were injected with both Pluronic to inhibit lipolysis and ^35^S-labeled methionine/cysteine (Met/Cys) to label newly synthesized proteins over a 2-hour period. In multiple independent powered experiments (*n =* 4), we observed no difference in TG secretion over the 2-hour labeling period (Figure 1, B and C) or secretion of newly synthesized total apoB-100 at 60, 90, or 120 minutes after injection (Figure 1, D–F). Similarly, there was also no change in the secretion of other plasma proteins in the size range of albumin (alb), apoE, or apoA-I secretion (Figure 1G) or secretion of apoB-48 (Supplemental Figure 2A; supplemental material available online with this article; https://doi.org/10.1172/JCI144334DS1). Importantly, there was no difference in the distribution of secreted lipoproteins among the mice, indicating the particle size of newly secreted lipoproteins does not differ between the 2 groups (Figure 1, H and I).

To determine if hepatocyte-specific loss of *Sort1* differed from the global KO, we repeated the in vivo TG and total apoB-100 secretion studies in 2 different liver-specific mouse models. We injected *Sort1^fl/fl^* mice with AAV8.TBG.Cre to delete *Sort1* in hepatocytes or AAV8.TBG.null as controls. We separately crossed hemizygous Alb-Cre^+^-transgenic mice with *Sort1^fl/fl^* mice to delete *Sort1* in hepatocytes. Consistent with the global *Sort1* KO, we did not observe any difference in plasma lipids (Supplemental Table 1). Additionally, there was no difference in TG or apoB-100 secretion in either model as compared with their littermate controls in either model (Supplemental Figure 1). Given our observations that neither global *Sort1*-KO nor hepatocyte-specific *Sort1*-KO mice under chow-fed basal conditions had evidence for altered apoB-100 secretion, we chose to focus our subsequent experiments on the global *Sort1*-KO mice (see below).

### Hepatic overexpression of sortilin reduces TG secretion but not apoB-100 secretion in basal chow-fed mice.

**Figure 2:**
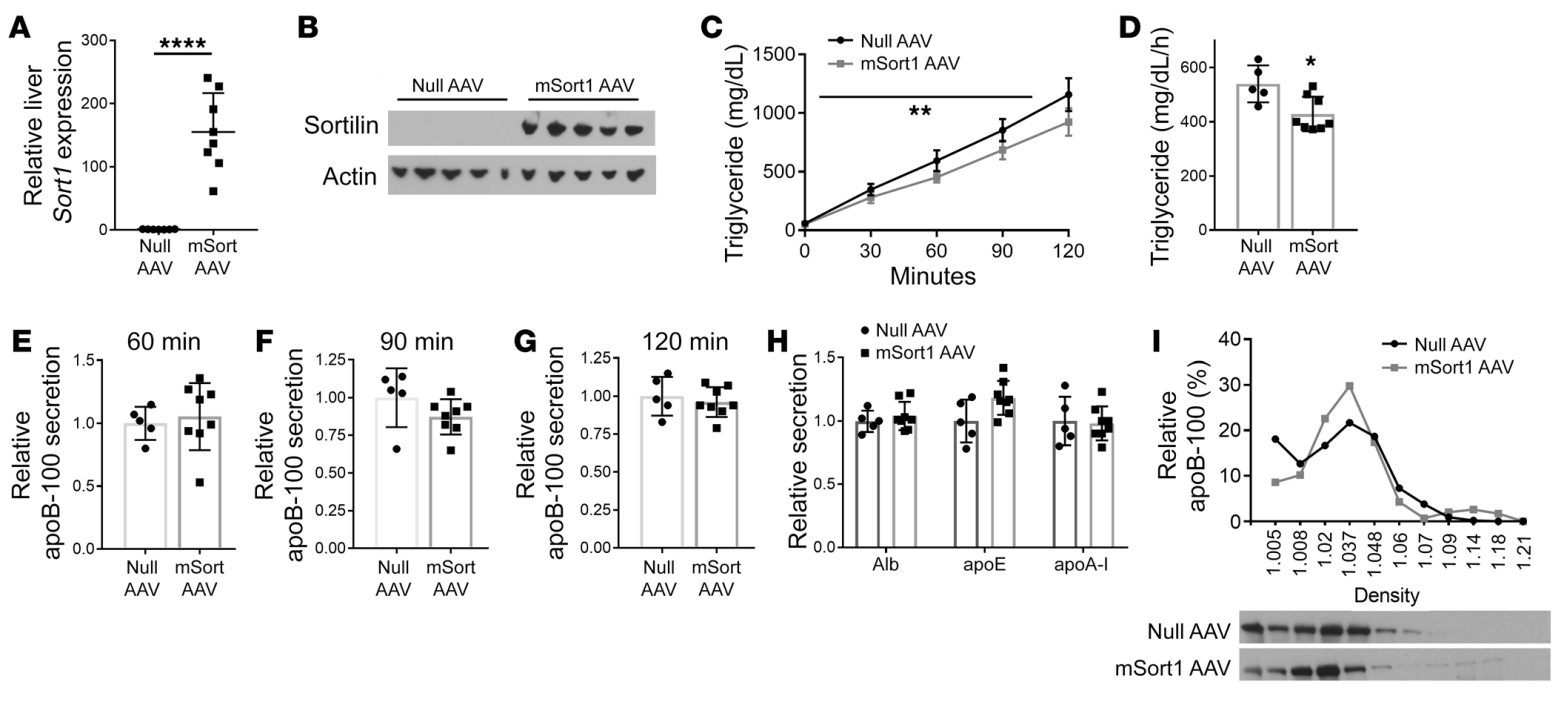
Hepatic overexpression of sortilin reduces TG but not apoB-100 secretion in basal chow-fed mice. 8-week-old C57BL/6J mice were injected with either null or mSort AAV.TBG and maintained on a chow diet for 12 weeks. (**A**) Hepatic *Sort1* mRNA was measured by qRT-PCR. ****P <0.0001 by *t* test. (**B**) Liver homogenate was Western blotted for sortilin and actin protein. (**C**) Mice were injected i.v. with Pluronic and ^35^S Met/Cys and bled every 30 minutes for 2 hours. Plasma TG was measured and compared using repeated-measures ANOVA. **P <0.005 by 2-way ANOVA. (**D**) TG secretion rate was calculated as the mg/dL/h. *P < 0.05 by *t* test. (**E**–**G**) Total plasma from each mouse at 60, 90, and 120 minutes after injection was subjected to autoradiography, and ^35^S-labeled apoB-100 was quantified by liquid scintillation counting. (**H**) Total plasma from the 2-hour postinjection time point was subjected to autoradiography, and ^35^S-labeled Alb, apoE, and apoA-I bands were counted. *n =* 8–9/group. (**I**) 2-hour plasma was pooled and separated by sucrose density ultracentrifugation and then taken off in 11 fractions, and total apoB-100 was measured by Western blotting. Data are presented as percentage of total apoB-100 in each fraction for each group. *P < 0.05 as compared with null AAV by Student’s *t* test.

Hepatic overexpression of sortilin has been consistently shown to reduce TG secretion in mice (11–13), but the effects on total apoB-100 secretion are much less well studied (13). To determine the effect of murine *Sort1* (mSort1) overexpression on total newly synthesized apoB-100, we injected an adeno-associated virus (AAV) vector expressing mSort1 or a control null AAV into 8-week-old chow-fed C57BL6/J mice. We noted a significant increase in hepatic sortilin mRNA and protein abundance (Figure 2, A and B). 12 weeks after injection, plasma cholesterol levels were significantly decreased, while there was no effect on plasma TG levels (Table 1). As above, the mice were injected with both Pluronic and ^35^S Met/Cys. As our lab (11) and others (13) have previously reported, there was a significant decrease in TG secretion in *mSort1*-overexpressing mice as compared with control mice (Figure 2, C and D). However, remarkably, there was no difference in the secretion of newly synthesized total apoB-100 in the plasma at 60, 90, or 120 minutes after injection (Figure 2, E–G). There was also no difference in alb, apoE, apoA-I, or apoB-48 secretion, as controls (Figure 2H and Supplemental Figure 2B).

Because there was a dissociation between the effects of *Sort1* overexpression on TG versus apoB-100 secretion, we used plasma from the 2-hour postinjection bleed to perform sucrose density gradient separation of lipoproteins. We noted that the *Sort1*-overexpressing mice had a marked decrease in newly synthesized apoB-100 in the VLDL fraction but a corresponding increase in the LDL fractions (Figure 2I). This is consistent with an effect of *Sort1* overexpression reducing the secretion of TG-rich VLDL, resulting in a shift to more apoB-100 being secreted in denser TG-poor LDL particles, with no effect on total apoB-100 secretion. This suggests that measurement of total apoB-100 secretion, as opposed to only VLDL apoB-100 secretion, may be important to understand the overall effect of sortilin’s regulation of apoB-100 secretion.

### Loss of sortilin has no effect on apoB-100 secretion in primary murine hepatocytes, HepG2 cells, or McA-RH7777 rat hepatoma cells under basal conditions.

**Figure 3:**
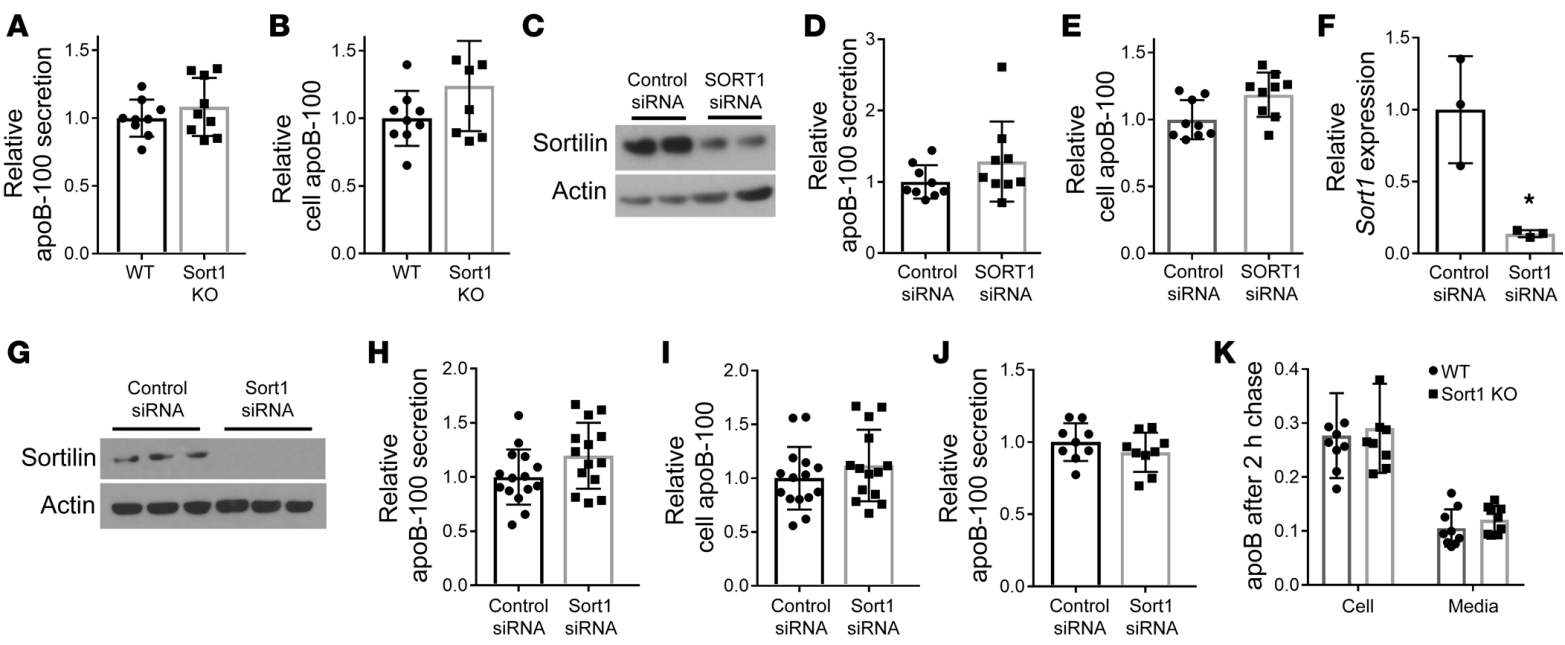
Loss of sortilin has no effect on apoB secretion in primary murine hepatocytes ex vivo, HepG2 cells, or McA-RH7777 rat hepatoma cells under basal conditions. (**A** and **B**) Primary hepatocytes were isolated from *Sort1*-KO mice and their WT littermates, and, 2 hours after plating, they were incubated for 1 hour without Met/Cys and then labeled in the same media with ^35^S Met/Cys for 2 hours. Media was collected, and cells were lysed, immunoprecipitated with apoB antibody, and subjected to autoradiography. *n =* 24 wells from 4 mice per group. (**C**) HepG cells were transfected with *SORT1* siRNA or control siRNA. After 36 hours, cells were lysed and Western blotted for sortilin and actin protein. (**D** and **E**) ApoB-100 secretion was measured, as described for **A** and **B**. *n =* 9/group from 3 independent experiments. (**F**) McA cells were transfected with rat *Sort1* (r*Sort1*) siRNA or control siRNA. After 36 hours cells were collected for RNA extraction, and *Sort1* mRNA was measured by qRT-PCR. *P < 0.05 by *t* test. (**G**) Other cells were lysed and separated by gel electrophoresis and Western blotted for sortilin and actin protein. Representative of 3 independent transfections. (**H** and **I**) Newly synthesized apoB-100 secretion was measured in siRNA-transfected McA cells, as described in **A** and **B**. *n =* 15 wells/group from 5 independent experiments. (**J**) siRNA-transfected McA cells were incubated for 1 hour without Met/Cys and then labeled in the same media with ^35^S Met/Cys for 20 minutes and chased for 10 minutes. Media was collected, and cells were treated as described above. (**K**) Additional wells were incubated in the chase media for 2 hours and similarly processed. Data are presented as relative newly synthesized apoB-100 from the 10-minute time point remaining in the cell or media. *n =* 9 wells per group per time point from 3 independent experiments.

In light of our observation that neither deletion nor overexpression of sortilin had an effect on total apoB-100 secretion in chow-fed mice under basal conditions in vivo, we turned to ex vivo and in vitro models to confirm this observation. We isolated primary hepatocytes from *Sort1*-KO mice and their littermate controls, labeled the cells with ^35^S Met/Cys for 2 hours to label newly synthesized proteins, and measured the appearance of total apoB-100 in the media. We found no difference in apoB-100 secretion or intracellular apoB-100 between *Sort1*-KO hepatocytes and those from WT littermate controls, indicating that the cells were secreting the same number of particles (Figure 3, A and B). Interestingly, there was an increase in TG secretion that was not observed in the *Sort1*-KO mice in vivo, and sucrose density gradient ultracentrifugation showed that larger particles were being secreted (Figure 3, A and B). To ensure the difference in particle number secretion was not a rodent-specific effect, we knocked down *SORT1* in HepG2 cells (Figure 3C) and found no difference in apoB-100 secretion or cellular apoB-100 with loss of *SORT1* expression (Figure 3, D and E).

We then turned to a well-established rat hepatoma cell line, McA-RH7777 (McA), for further investigation. The regulation of apoB-100 secretion in response to various stimuli by both ERAD and PERPP pathways has been well studied in McA cells by multiple groups (19, 23, 27). We treated McA cells with an siRNA against *Sort1*, which reduced *Sort1* mRNA by 90% and could not detect sortilin protein remaining in the cells after 36 hours (Figure 3, F and G). At this time, the cells were labeled with ^35^S Met/Cys, and then media and cells were harvested. We found that neither apoB-100 secretion nor cellular apoB-100 levels were affected in *Sort1*-knockdown cells as compared with control cells (Figure 3, H and I). Similarly to primary hepatocytes, there was a significant increase in TG secretion, with loss of *Sort1* expression once again demonstrating a particle size difference, despite no change in particle number (Supplemental Figure 3C). To confirm that the absence of effect on apoB-100 secretion with knockdown of *Sort1* was not due to any other alterations in apoB-100 metabolism, we performed pulse-chase studies in which McA cells were pulse labeled with ^35^S Met/Cys for 20 minutes and then chased in unlabeled media for 2 hours. *Sort1* siRNA–treated cells had no difference in the amount of newly synthesized apoB-100 produced or apoB remaining in the cell or media after the 2-hour chase, indicating that there were no differences in either synthesis of apoB-100 or its degradation over the 2-hour period (Figure 3, J and K). Based on multiple models, we conclude that, in a basal state, loss of sortilin does not affect total apoB-100 secretion, consistent with our in vivo observations in mice.

### In cells overexpressing human apoB-100 or treated with fatty acids, knockdown of sortilin results in a significant increase in total apoB-100 secretion.

**Figure 4:**
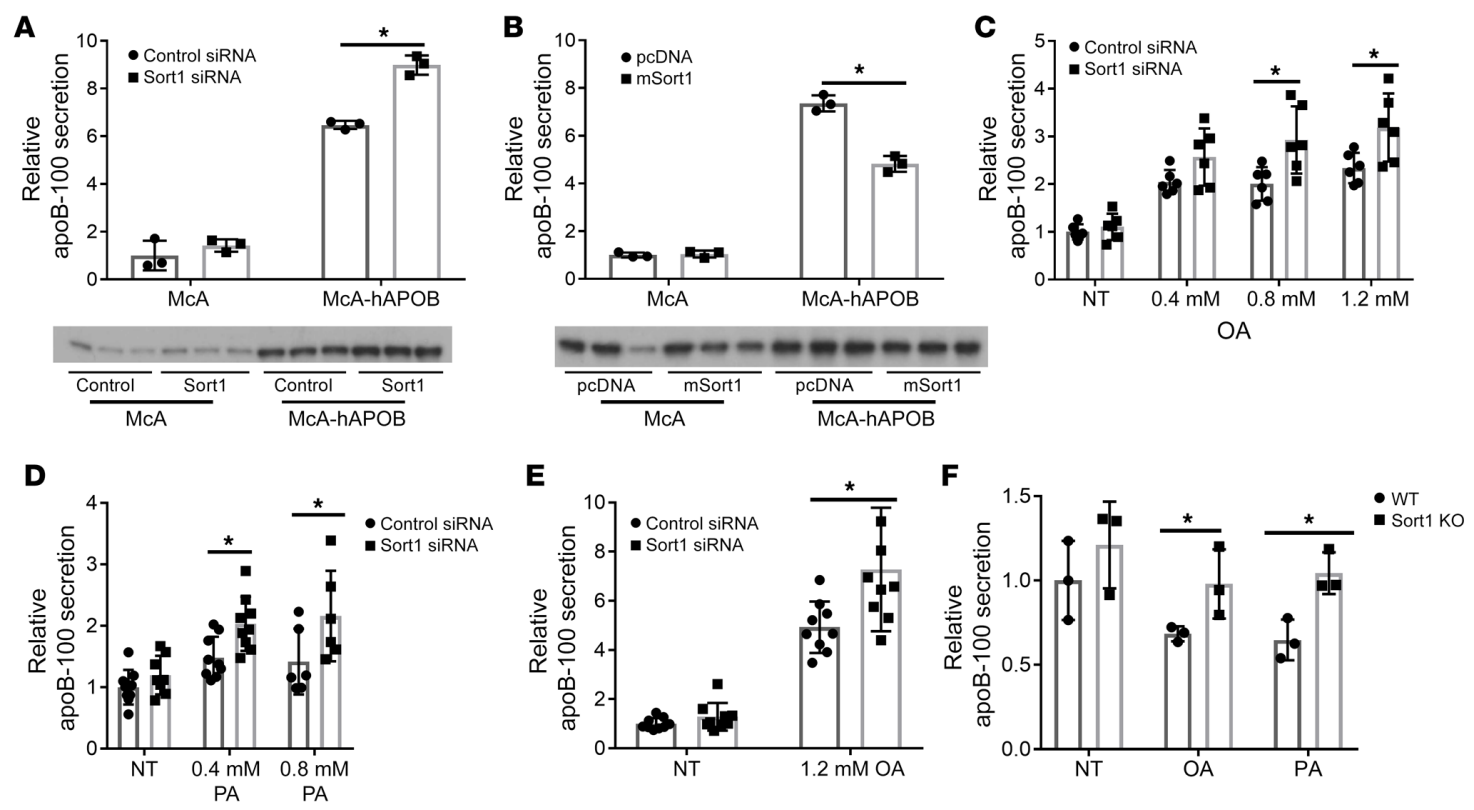
In cells overexpressing human apoB or treated with fatty acids loss of sortilin results in a significant increase in apoB-100 secretion. (**A**) McA and McA-HAPOB cells were transfected with rat *Sort1* (r*Sort1*) siRNA or control siRNA. After 36 hours, cells were incubated for 1 hour without Met/Cys and then labeled in the same media with ^35^S Met/Cys for 2 hours. Media was collected and cells were lysed and subjected to autoradiography, and ^35^S-labeled apoB-100 was quantified by liquid scintillation counting counted. *n =* 3 wells/group. Representative of 3 independent experiments. (**B**) McA and McA-HAPOB cells were transfected with m*Sort1* or empty control vector (pcDNA), and apoB-100 secretion was measured as described in **A**. *n =* 3 wells/group. Representative of 3 independent experiments. (**C**) siRNA-transfected McA cells were incubated without Met/Cys with 0(NT), 0.4, 0.8, or 1.2 mM oleic acid (OA) for 2 hours and then labeled in the same media with ^35^S Met/Cys for 2 hours, after which apoB-100 secretion was measured. *n =* 9 wells/treatment/group. (**D**) apoB secretion from McA cells treated with 0, 0.4 mM, or 0.8 mM palmitic acid (PA). *n =* 9 wells/treatment/group. (**E**) apoB secretion in siRNA-transfected HepG2 cells treated with 0 or 1.2 mM OA. *n =* 9 well/treatment/group. (**F**) apoB secretion in primary hepatocytes isolated from *Sort1*-KO mice and WT littermate controls treated with 0.4 mM OA or 0.4 mM PA or nontreated (NT) for 4 hours. *n =* 3 wells/mouse. Representative of 3 independent isolations. *P < 0.05 versus control siRNA or pcDNA by ANOVA.

In contrast to the lack of effect of sortilin deficiency on apoB-100 secretion in WT mice and primary hepatocytes, we had previously reported that, in primary hepatocytes from *hapoB-Tg;Apobec1^–/–^* mice, treatment with an siRNA against *Sort1* resulted in increased apoB-100 secretion (11). This suggested the possibility that the markedly increased synthesis of apoB-100 in the livers of these mice might unmask an apoB-100 secretion phenotype associated with loss of *Sort1* expression. We tested this hypothesis in a McA cell line with *hAPOB* stably overexpressed (McA-hAPOB cells; ref. 30). We confirmed that apoB-100 secretion was increased over 6-fold as compared with WT McA cells (Figure 4A). After treating McA-hAPOB cells with an siRNA against *Sort1*, there was a significant increase in apoB-100 secretion as compared with that in McA-hAPOB cells treated with a control siRNA, despite no difference in cellular apoB-100 (Figure 4A and Supplemental Figure 4A). There was a significant decrease in TG secretion in the McA-hAPOB cells as compared with WT McA cells, indicating that these cells secret smaller TG-depleted apoB-100 containing particles, as observed in the *hapoB-Tg;Apobec1^–/–^* mice, and, under these conditions, we did not observe a difference in TG secretion (Supplemental Figure 3E).

To see if a reciprocal relationship existed, we transfected McA and McA-hAPOB cells with a vector overexpressing *mSort1* or an empty vector control plasmid (pcDNA). In the McA cells, overexpression of *Sort1* had no effect on apoB-100 secretion. However, in McA-hAPOB cells, overexpression of *Sort1* resulted in a significant decrease in apoB-100 secretion (Figure 4B). There was no difference in total cellular apoB-100 between the groups under any condition (Supplemental Figure 4b)

To discern whether the differences we observed between the 2 mcA cell lines were due to an increase in relative apoB-100 secretion, we sought to increase apoB secretion in a more physiologic manner by acutely lipid-loading McA cells with 0.4, 0.8, or 1.2 mM oleic acid (OA) for 4 hours (31). *Sort1* knockdown had no effect on apoB-100 secretion in the absence of OA, but under treatment with 0.8 mM and 1.2 mM OA, *Sort1* knockdown significantly increased apoB-100 secretion (Figure 4C). Similar results were seen with palmitic acid (PA), with an increase in apoB-100 secretion in the *Sort1*-knockdown cells starting at lower doses of 0.4 mM and 0.8 mM (Figure 4D). The increase in apoB-100 secretion was specific, as there was no effect on either cellular apoB-100 or newly synthesized alb or apoB-48 secretion (Supplemental Figure 4, C and D, and Supplemental Figure 5A). To ensure that this was not a rodent-specific effect, HepG2 cells were treated with 1.2 mM OA and a *SORT1* siRNA; the OA-treated cells showed increased apoB-100 secretion (Figure 4E). Once again, there was no difference in cell apoB-100 levels or alb secretion (Supplemental Figure 4E and Supplemental Figure 5B). *Sort1*-KO primary hepatocytes also demonstrated an increase in apoB-100 secretion with treatment of 0.4 mM OA or PA compared with WT littermate hepatocytes (Figure 4F).

### High-fat diet feeding unmasks an effect of Sort1 deletion or overexpression on apoB-100 secretion in mice.

**Figure 5:**
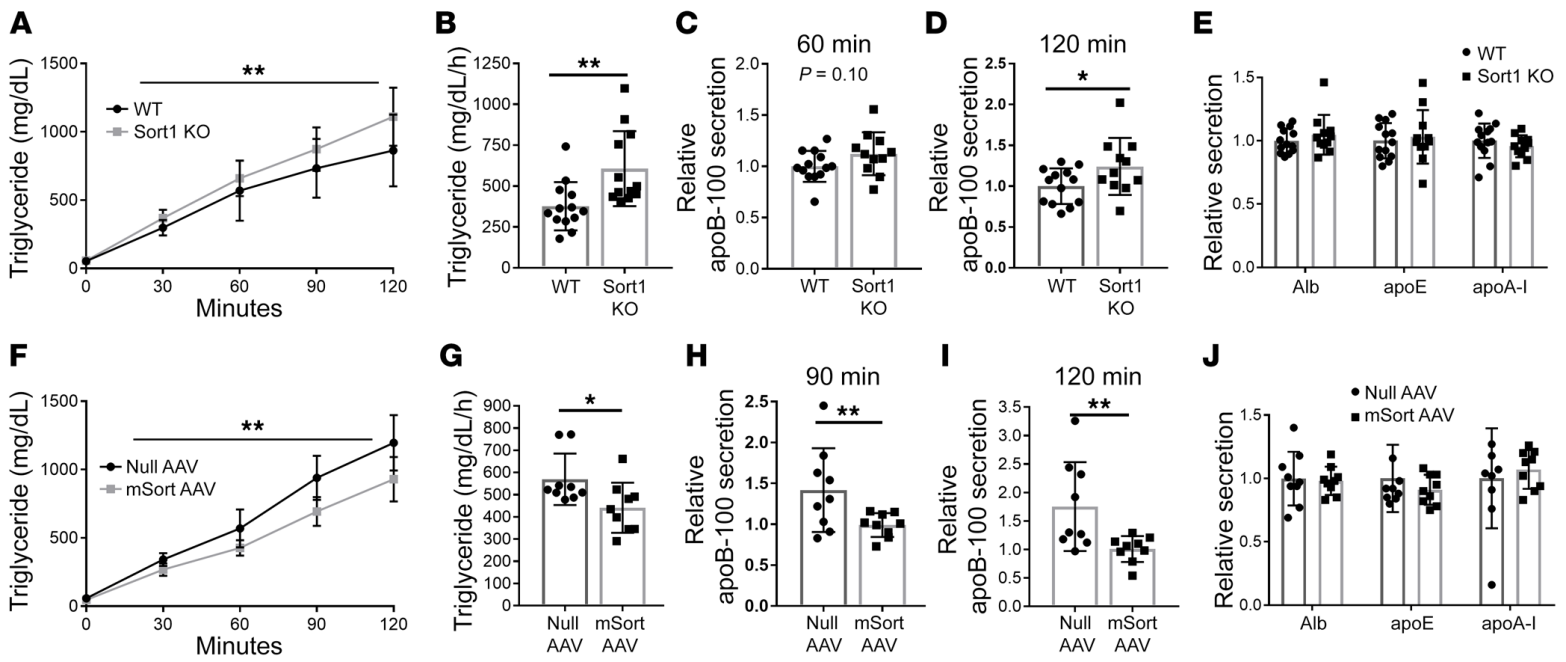
Feeding mice a high-fat diet unmasks an effect of Sort1 deletion or overexpression on apoB-100 secretion. (**A**) 8- to 10-week-old WT and *Sort1*-KO mice were placed on a 45% high-fat diet for 12 weeks. Mice were injected i.v. with Pluronic and ^35^S Met/Cys and bled every 30 minutes for 2 hours. Plasma TG was measured and compared using repeated-measures ANOVA. **P < 0.01 by 2-way ANOVA. (**B**) TG secretion rate was calculated as the mg/dL/h. **P < 0.01 by *t* test. (**C** and **D**) Total plasma from each mouse at 60 and 120 minutes after injection was subjected to autoradiography, and ^35^S-labeled apoB-100 was quantified by liquid scintillation counting counted. (**E**) ^35^S-labeled Alb, apoE, and apoA-I bands were also counted in 120-minute total plasma. *n =* 12–14/group. *P < 0.05 as compared with WT by Student’s *t* test. (**F** and **G**) 8-week-old C57BL/6J mice were injected with either null or mSort AAV.TBG and maintained on a 45% high-fat diet for 12 weeks. Mice were injected i.v. with Pluronic and ^35^S Met/Cys and bled every 30 minutes for 2 hours. TG secretion and rate was as described in **A** and **B**. **P < 0.01 by 2-way ANOVA. (**H** and **I**) ^35^S-labeled apoB-100 was quantified by liquid scintillation counting at 60 and 120 minutes as described above. **P < 0.01 by *t* test. (**J**) ^35^S-labeled Alb, apoE, and apoA-I protein after 120 minutes was measured as described above. *n =* 9–10group. *P < 0.05 as compared with null AAV by Student’s *t* test.

As shown above, neither *Sort1* deletion nor overexpression in mice fed a chow diet affected total apoB-100 secretion. Based on our cellular studies, we hypothesized that excess hepatocyte lipid may reveal an effect of sortilin deficiency or overexpression on apoB-100 secretion. Therefore, we placed *Sort1*-KO mice on a 45% high-fat diet (HFD) for 12 weeks. Body weights trended higher in the *Sort1*-KO mice compared with WT mice, but the differences were not statistically significant (Table 1). Plasma cholesterol levels were significantly higher by 20% in the *Sort1*-KO mice compared with WT mice, while plasma TG levels were not different (Table 1). We performed a TG/apoB-100 production study after 12 weeks of HFD and observed significant increases in TG secretion (Figure 5, A and B) and total apoB-100 secretion (Figure 5, C and D) in the *Sort1*-KO mice compared with WT littermate controls. *Sort1*-KO mice had no change in secretion of other plasma proteins, including apoB-48, alb, apoE, and apoA-I (Figure 5E and Supplemental Figure 2C). The increase in apoB-100 secretion was not due to the increased age of the mice on HFD, as age-matched *Sort1*-KO and WT mice maintained on a chow diet did not show any differences in secretion (Supplemental Figure 6).

We also asked whether HFD feeding influenced the effect of *Sort1* overexpression on apoB-100 secretion. WT mice injected with AAV-*mSort1* or control vector (AAV-null) were placed on a 45% HFD for 12 weeks. Body weights were not different between the 2 groups. Plasma cholesterol levels were significantly lower by 43% in the *Sort1*-overexpressing mice compared with control mice, and plasma TG levels were also significantly lower by 21% (Table 1). We performed a TG/apoB-100 production study at 12 weeks of diet and observed significant decreases in TG secretion (Figure 5, F and G). While chow-fed mice overexpressing *Sort1* had no change in total apoB-100 secretion (Figure 2), the mice fed HFD and overexpressing *Sort1* had a significant decrease in total apoB-100 secretion (Figure 5, H and I) and apoB-48 secretion (Supplemental Figure 2D), but no increase in other secreted proteins was seen (Figure 5J). We concluded that dietary lipid overload results in an unmasking of the effects of sortilin in the regulation of apoB-100 secretion in a manner that was not observed in basal chow-fed conditions.

### Induction of hepatic ER stress also reveals an effect of sortilin on apoB-100 secretion.

**Figure 6:**
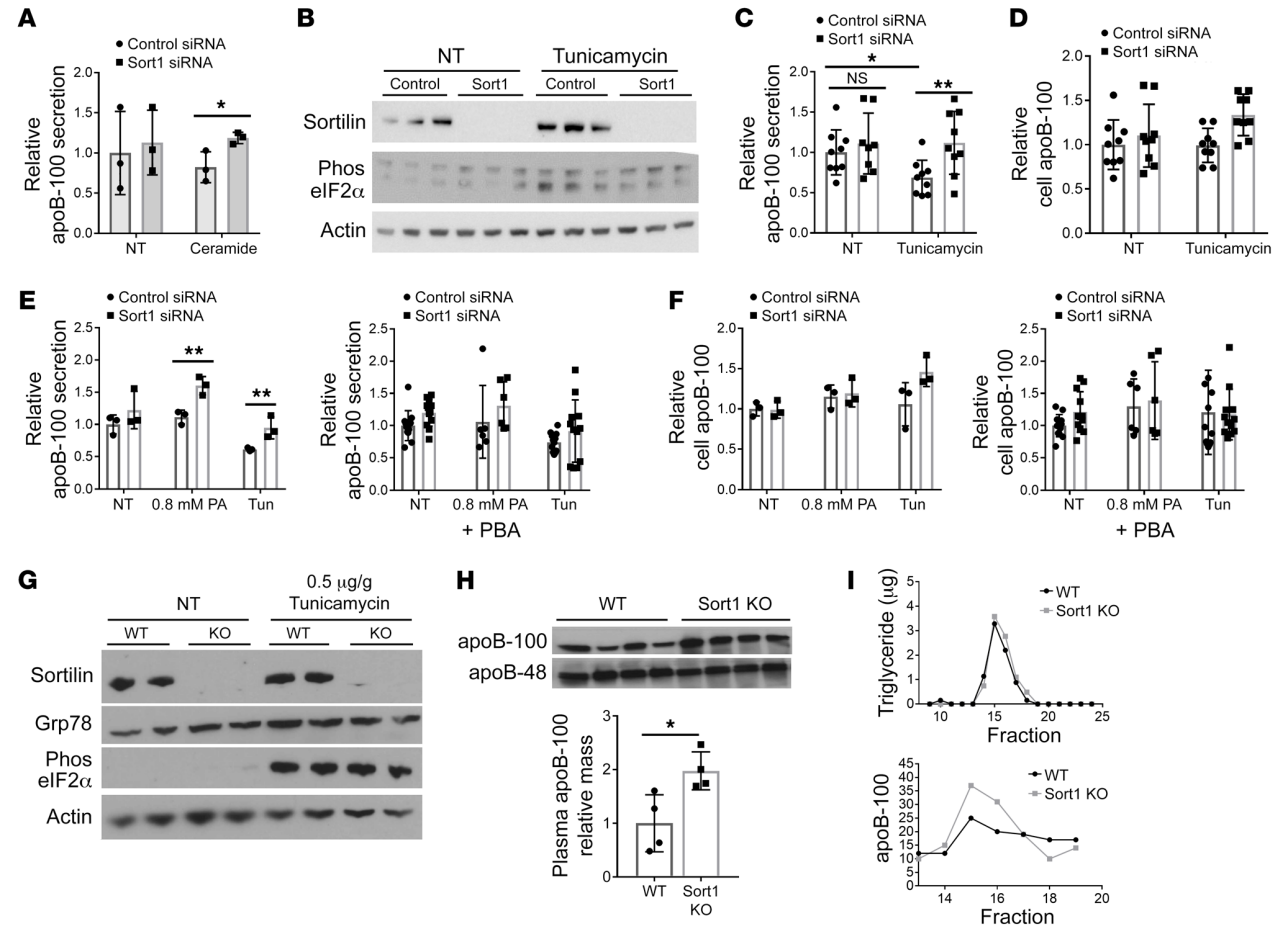
Induction of hepatic ER stress also reveals an effect of sortilin on apoB-100 secretion. (**A**) siRNA-transfected McA cells were incubated without Met/Cys with 0 (NT) or 10 μM ceramide for 2 hours and then labeled in the same media with ^35^S Met/Cys for 2 hours, after which apoB-100 secretion was measured. *n =* 6 wells/treatment/group. (**B**) siRNA-transfected McA cells were treated with 1 μM tunicamycin for 4 hours, and then cells were lysed and Western blotted for sortilin, Grp78, phos-eIF2a, and actin protein. Representative blots from 3 experiments. (**C** and **D**) Newly synthesized apoB-100 secretion and cell apoB-100 were measured in tunicamycin-treated (1 μM) siRNA-transfected McA cells as in **A**. *n =* 9 wells/group/treatment from 3 experiments. (**E** and **F**) McA cells were treated with 0.8 mM PA or 1 μM tunicamycin with or without the addition of 4-phenylbutyric acid (PBA). Newly synthesized apoB100 was measured in media (**E**) and cells (**F**). (**G**–**I**) WT and Sort1-KO mice were injected i.p. with 0.05 μg/g body weight tunicamycin. After 4 hours, mice were injected with Pluronic and bled after 2 hours. (**G**) Livers were homogenized and Western blotted for sortilin, Grp78, phos-eIF2a, and actin protein. (**H**) 2 hours after Pluronic plasma was immunoblotted for apoB and quantified by densitometry. (**I**) Pooled 2 hour plasma was separated by FPLC, and TG and apoB100 were measured. *P < 0.05, **P < 0.01 by ANOVA.

An increase in apoB-100 secretion in the absence of *Sort1* expression was observed at a lower dose of PA than OA in McA cells as well as in primary hepatocytes. While these fatty acids increase apoB-100 secretion, they are also associated with increased levels of ER stress, PA to a greater extent than OA (32). We observed the presence of elevated ER stress markers in both the PA- and OA-treated primary hepatocytes as compared with untreated and PA-treated McA cells but not OA-treated cells (Supplemental Figure 7). The regulation of apoB secretion under ER stress conditions is unique and complex (19). Increased apoB itself in the ER is sufficient to induce ER stress in hepatocytes (33). Lipid-induced ER stress first results in an increase in apoB secretion as an adaptive response to protect the ER from excess lipid (31). Prolonged or severe ER stress from lipids and other causes ultimately leads to a decrease in apoB secretion from the hepatocyte, as a portion of the apoB is targeted for degradation through an undefined pathway that is not solely ERAD (31, 32). We hypothesized that sortilin may be involved in the apoB-related ER stress response with regard to apoB secretion and that induction of ER stress may reveal an effect of sortilin knockdown or deletion on apoB secretion. In order to separate the effects of FAs from the induction of ER stress, we treated McA cells with known inducers of ER stress and compared apoB-100 secretion in cells after siRNA knockdown of *Sort1* compared with controls. As noted above, knockdown of *Sort1* in untreated basal McA cells had no effect on apoB secretion (Figure 6A). In the setting of treatment with 10 μM ceramide, which is elevated with PA treatment and known to increase ER stress (32, 34), *Sort1* knockdown resulted in a significant increase in apoB-100 secretion (Figure 6A and ref. 31). These results suggest that the effect of sortilin on apoB-100 secretion may be dependent on the amount of apoB-100 that exits the ER, influenced by the degree of ER stress.

We next treated McA cells with 1 μM tunicamycin to induce ER stress and confirmed an increase in markers of ER stress, Grp78 and phos-eIF2a (Figure 6B). Importantly, this treatment did not decrease sortilin protein in the control siRNA-treated cells (Figure 6B), as ER stress has previously shown to decrease *Sort1* transcription (13). As previously reported (31, 35), apoB-100 secretion was decreased in the control cells in response to tunicamycin. However, in the absence of *Sort1*, this decrease in apoB-100 secretion in response to ER stress induction was prevented and therefore higher than in control cells (Figure 6C), with no changes in cellular apoB-100 levels (Figure 6D), indicating that the increased secretion was not due to a change in apoB-100 synthesis. To determine whether the inhibition of ER stress would prevent the increase in apoB-100 secretion, we pretreated siRNA-transfected McA cells with or without 4-phenylbutyric acid (PBA) and then treated the cells with 0.8 mM PA or 1 μM tunicamycin. The *Sort1* siRNA–transfected cells that were not treated with PBA showed increased apoB-100 secretion in the PA- and tunicamycin-treated groups, while the cells with PBA did not demonstrate any difference in apoB-100 secretion with any treatment (Figure 6, E and F). There was no change in apoB-100 levels in the cells of any treatment group, indicating a secretion specific effect (Figure 6F). These data demonstrate that the inhibition of ER stress eliminated the sortilin effect on apoB-100 secretion.

We then injected *Sort1*-KO and littermate controls with 0.05 μg tunicamycin per gram of body weight; 4 hours later, we injected the mice with Pluronic. Tunicamycin-treated mice had increased levels of hepatic Grp78 and phos-eIf2a protein, demonstrating that there was an increase in hepatic ER stress (Figure 6G). Sortilin protein levels were not decreased by this acute 0.05 μg/g tunicamycin treatment (Figure 6G). To confirm previous reports that showed a decrease in *Sort1* transcription in response to tunicamycin, we also injected mice with the higher doses of 0.5 and 1 μg/g of tunicamycin. At a 1 μg/g dose of tunicamycin, *Sort1* message levels were decreased, as previously reported, but sortilin protein levels were unchanged in the WT mice at all doses after 6 hours (Supplemental Figure 8, A and B). There was a significant decrease in TG secretion in both WT and *Sort1*-KO mice treated with tunicamycin, but only *Sort1*-KO mice had higher levels of total apoB-100 mass at all doses of tunicamycin (Figure 6H and Supplemental Figure 8, C and D). In mice treated with 0.05 μg/g tunicamycin, apoB-100 was elevated in the VLDL fraction in *Sort1*-KO mice compared with that in WT mice, despite no observed difference in VLDL-TG (Figure 6I), suggesting that there is an increase in VLDL particles with loss of *Sort1* expression. These results show that ER stress unmasks a role for sortilin in limiting apoB-100 secretion and that, in the setting of ER stress, the lack of sortilin results in more apoB-100 targeted for secretion.

### Carriers of rs12740374 with diabetes had a greater decrease in plasma apoB, TG, VLDL, and LDL compared with people without diabetes.

**Figure 7:**
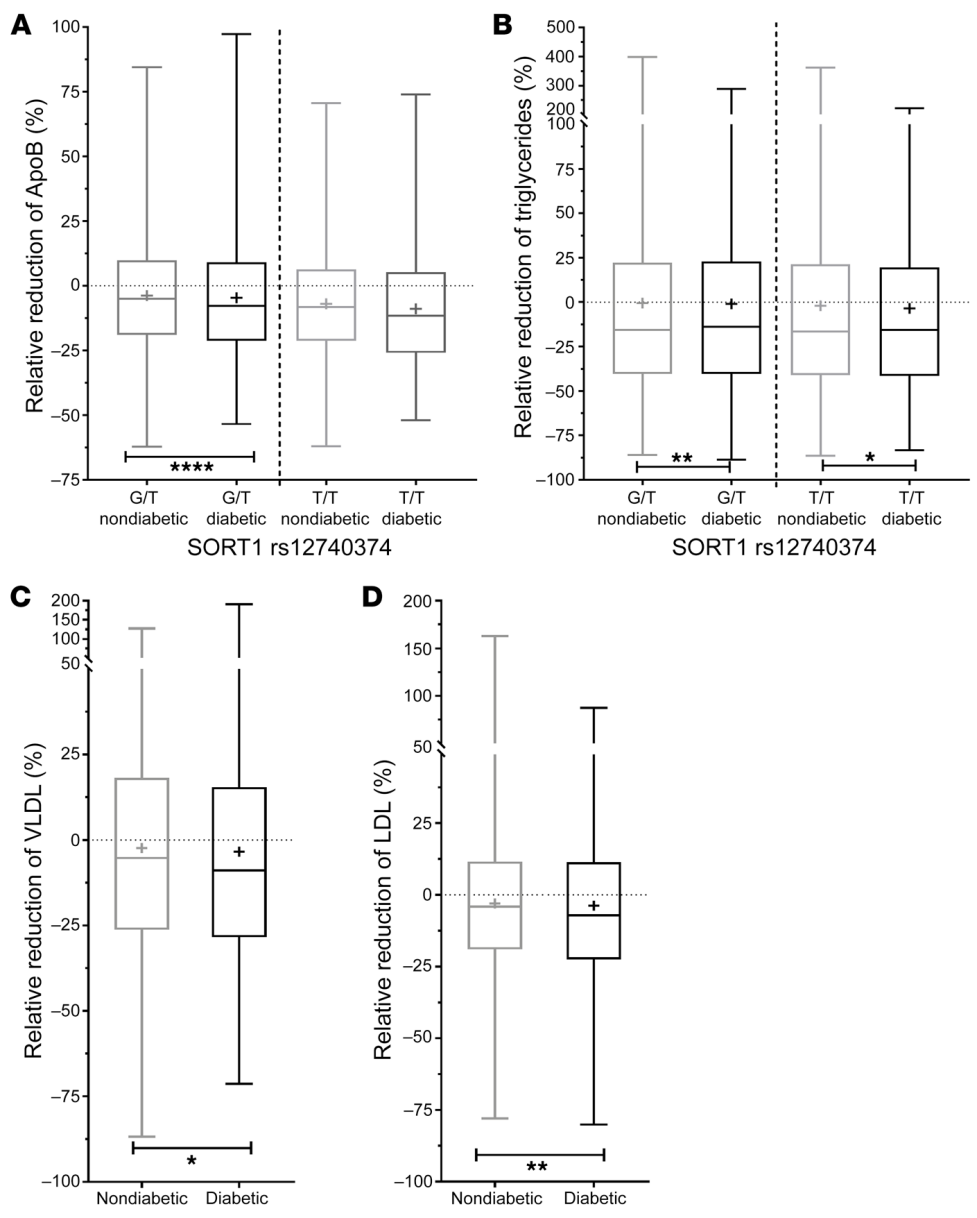
Carriers of rs12740374 with diabetes had a greater decrease in plasma apoB, triglyceride, VLDL, and LDL compared with people without diabetes. Relative reduction of (**A**) ApoB, (**B**) triglycerides, (**C**) VLDL, and (**D**) LDL in people with and without diabetes who are heterozygote carriers of SORT1 *rs12740374*. The center line indicates the median, while the box represents the 25th to 75th percentiles. The plus sign represents the mean for each group, and the whiskers mark minimum and maximum values. Asterisks indicate significant differences between people with diabetes and people without diabetes. *P < 0.05, **P < 0.01, ****P < 0.0001 by multivariable regression adjusted for age, sex and BMI. Number of people with and without diabetes who are rs12740374 carriers: G/G, 261,393/14,100; G/T, 149,001/7,274; and T/T, 21,277/1201.

Carriers of the rs12740374 variant, which is associated with increased hepatic sortilin expression, have been consistently shown to have decreased LDL-C and apoB levels, but the relative decrease has not been compared in a metabolic stressed condition, such as the presence of type 2 diabetes. In order to determine if the same relationship observed in cells and mice also existed in humans, we compared the relative reduction in apoB and TG levels in carriers of the rs12740374 variant with diabetes with that in carriers without diabetes in almost 500,000 individuals in the UK Biobank. There was a significantly greater decrease in both apoB and TG in carriers of the rs12740374 variant with diabetes (Figure 7, A and B). While plasma apoB measurements were not available, we were able to replicate the observed decrease in plasma TG in rs12740374 carriers with diabetes as compared with people without diabetes in our internal Penn Medicine Biobank (data not shown). Metabolomics analysis performed in a subset of this samples in the UK Biobank revealed a decrease in the concentration of VLDL and LDL particles. The decrease in apoB-containing particle number was also greater in the carriers of rs12740374 with diabetes (Figure 7, C and D). All together, these analyses suggest that the rs12740374 variant, which increases sortilin expression in the liver, has a greater effect on apoB-lipoprotein production in the metabolically stressed diabetic state compared with the nondiabetic state.

## Discussion

The experiments described here sought to resolve the conflicting data that has arisen in the literature over the past 10 years about the directionality of sortilin’s effect on apoB-100 secretion, a topic that has been the subject of numerous reviews (28, 36, 37). We show conclusively that under basal chow-fed conditions, neither global nor hepatocyte-specific *Sort1* deletion in mice influenced total apoB-100 secretion in vivo, results that were confirmed by in vitro studies of several hepatocytic cell types under basal conditions. In contrast, fatty acid treatment and other conditions that increase ER stress in hepatocytes unmasked an effect of sortilin on apoB-100 secretion; under these conditions, deletion of sortilin increased apoB-100 secretion, while overexpression of sortilin decreased apoB-100 secretion. Finally, in distinct contrast to a chow diet, treatment of *Sort1*-KO mice with a HFD resulted in a significant increase in apoB-100 secretion, with a reciprocal effect of *Sort1* overexpression. We conclude that, under basal non-ER-stressed conditions, deletion of sortilin has little effect on hepatocyte apoB-100 secretion, but that, in the setting of lipid loading or induced ER stress, reduced hepatic sortilin leads to increased apoB-100 secretion and increased sortilin leads to decreased apoB-100 secretion (Figure 7), a directionality of effect completely consistent with the human genetics data.

Kjolby et al. (16) originally reported, in a different *Sort1*^–/–^ mouse (38), that after tyloxapol injection, apoB-100 mass in plasma, as assessed by Western blotting, was lower in *Sort1*^–/–^ mice relative to controls. However, total apoB-100 mass did not increase as expected with tyloxapol injection after 2 hours compared with preinjection apoB-100 mass, indicating the limitations of using mass for dynamic apoB-100 secretion studies. In contrast, the preferred method is to measure newly synthesized apoB-100 secretion using endogenous labeling, which is a more accurate reflection of secretion of apoB-100 and has been widely used for 3 decades (39–44). This is especially important, as apoB-100 mass is present not only in VLDL but also in LDL; furthermore, sortilin has been shown to play a role in LDL clearance (45, 46). Indeed, when Kjolby and colleagues measured newly synthesized apoB-100 after metabolically labeling primary hepatocytes, their results after 1 or 4 hours of chase are consistent with our reported results, as they did not observe a difference in secretion in basal conditions (16). The reported decrease in apoB-100 secretion occurred only after a very long chase of 12 or 20 hours, at which time newly synthesized alb was also reduced, suggesting that the dedifferentiation of the plated primary hepatocytes could also be a confounding factor. We also point out that the *Sort1*^–/–^ mouse model used in these experiments was protected from diet-induced obesity, was more insulin sensitive, and had decreased hepatic steatosis, as shown in a report from an another group (47). This phenotype differs from another *Sort1*-KO mouse model (48) as well as what we observed here in a third *Sort1*-KO model, in which HFD-fed mice had no difference in body weight (Table 1). ApoB-100 secretion can be influenced by extrahepatic effects, such as hepatic insulin signaling and decreased lipid delivery to the liver (49). Sparks and colleagues demonstrated that changes in insulin sensitivity in McA cells altered the effect of *Sort1* knockdown on apoB secretion (50). The differences in metabolic responses to HFD in *Sort1*-KO mice makes interpretation of the conflicting results more difficult, especially considering the role that *Sort1* plays in adipose tissue metabolism (51–53). Given the liver-specific effect of the variants at the *SORT1* locus on *SORT1* expression, we focused our studies on hepatocytes in an effort to eliminate any potential extrahepatic effects of *SORT1* deletion on apoB-100 secretion.

An important additional issue is the critical difference between measurements of VLDL-apoB-100 versus total apoB-100 secretion. While overexpression of hepatic *Sort1* in mice has shown a reduction in TG secretion, a careful analysis of these studies reveals that newly synthesized apoB-100 secretion was reported to be decreased when only apoB-100 in the VLDL fraction was measured (11, 12, 14, 15). Importantly, an abundance of data in mice and humans indicates that under certain conditions, the liver can secrete apoB-lipoproteins that are denser and less lipidated than classic VLDL (44, 54, 55), including in the hAPOB-transgenic mice and rabbits, where the majority of TG exists in the LDL fraction (56–58). The practical implication of this observation is that a focus solely on VLDL-apoB secretion can miss apoB-100 that is being secreted in denser particles. In studies where total newly synthesized apoB-100 in the plasma is measured, increased hepatic sortilin was associated with a decrease in apoB-100 secretion in *ob/ob* mice as well as in a HFD-fed mouse model (13). We observed here that C57BL/6J mice injected with *mSort1* AAV on a chow diet had no difference in total apoB-100 secretion but did have a reduction in VLDL-apoB-100 secretion, indicating a shift of apoB-100 secretion into denser particles. When these *mSort1*-overexpressing mice were placed on a HFD, there was then a significant decrease in total apoB-100 secretion. Additionally, overexpression of apoB itself has been shown to induce increased misfolding of apoB in the ER and induction of ER stress (59) as well as to affect the distribution of apoB-100 secretion into VLDL versus denser particles (56, 57). Our results in McA hepatoma cells with and without *hAPOB* expression show clear differences in apoB-100 secretion in response to knockdown or overexpression of *Sort1*, confirming that overexpression of *hAPOB* alters the physiological interaction of apoB-100 and sortilin. These cumulative results establish that it necessary to measure secretion of total newly synthesized apoB-100 as well as carefully consider conditions such as different mouse models and diets that may affect the physiological interaction of sortilin with apoB-100.

There has been much investigation into the regulation of apoB-100 secretion both co- and posttranslationally in the ER and its regulation in post-ER compartments, referred to as PERPP (19, 21, 22). The regulation of apoB-100 in response to the induction of ER stress has been shown to be complex due in part to the nature of the apoB-100 protein size and folding (31, 32). Induction of ER stress causes the misfolding of apoB, leading to it being targeted for degradation (60) through both proteasomal and autophagy pathways (60). Amengual and colleagues demonstrated that sortilin mediates the degradation of apoB-100 through the induction of autophagy and showed colocalization of apoB-100 with sortilin in autophagic vacuoles (15). We hypothesized that misfolded apoB-100 that exits the ER is more likely to interact with sortilin in the Golgi and, thus, more likely to be targeted for degradation by the autophagy/lysosomal pathway. Our data showed that knockdown of sortilin in the hepatocyte increased apoB-100 secretion under conditions that induce ER stress, consistent with the model that sortilin serves as a quality control step that results in increased degradation of apoB-100 and decreased total apoB-100 secretion. Hepatic *Sort1* transcription and sortilin protein are both decreased in insulin-resistant and obese mice by a variety of pathways, including changes in insulin signaling and increased ER stress response pathways (13, 14, 17, 53, 61), concurrent with increases in TG and apoB secretion (49). Thus, downregulation of hepatic sortilin may be a protective mechanism to promote unloading of lipid from the liver under stress conditions, while, at the same time, having the effect of increasing plasma apoB-containing lipoproteins (15, 19, 23–25). Our data also indicate that the mechanism by which sortilin regulates particle number and particle size differs, suggesting sortilin may also play a role in lipidation of the apoB-100 particle, which is an important area for future study.

Importantly, our proposed model is consistent with the directionality predicted by human genetics. Several SNPs, including rs12740374, that are associated with increased hepatic- specific *SORT1* expression are associated with reduced LDL-C (5, 8) and apoB-100 levels (9, 10). Interestingly, a recent study reported that these *SORT1* SNPs were more protective against cardiovascular disease risk in people with type 2 diabetes compared with those without type 2 diabetes mellitus (62). Our results suggest that this protection may be due to a decrease in VLDL production that leads to reduced apoB containing lipoproteins in carriers of the *SORT1* variants. This is consistent with a model in which hepatic *SORT1* plays a more important physiological role in the context of metabolic stress. Our studies indicate that hepatic sortilin inversely regulates the secretion of apoB-100 specifically under conditions of lipid loading and ER stress. They provide clarity about the directionality of sortilin in the regulation of hepatic apoB-100 and suggest that the complex interaction of sortilin and apoB-100 in the hepatocyte and the fate of newly synthesized apoB-100 are dependent on specific cellular conditions, including insulin resistance, lipid toxicity, and the presence of ER stress.

## Methods

### Animal models and care.

C57BL/6J mice and alb Cre^+^ mice were purchased from The Jackson Laboratory. *Sort1*-KO mice were a gift from Carlos Morales (McGill University, Montreal, Canada). *Sort1^fl/fl^* mice were a gift from Merck. All mice were housed 3–5 mice per cage in a climate controlled room with a 12:12-light/dark cycle with ad libitum access to regular chow diet. Where specifically noted in the text, animals were fed HFD ad libitum containing 45% kcal of fat (D12451, Research Diets) for a duration of 12 weeks. In some experiments, mice were injected with 0.05, 0.5, or 1 μg per gram of body weight of tunicamycin. Experiments were performed on 8– to 22-week-old male mice. Mice were assigned to experimental groups based on weight matching. Mice were euthanized by cervical dislocation after isoflurane inhalation.

### Cell lines.

McA-RH777 cells and HepG2 cells were obtained from ATCC. McA-hAPOB cells were a gift from Zemin Yao (University of Ottawa, Ottawa, Canada) (30). HepG2 cells were maintained in low-glucose DMEM (Invitrogen, 11885-084) containing penicillin (100 units/mL), streptomycin (100 μg/mL) (Invitrogen, 15140-122), and 10% FBS (Hyclone, SH30910.03). McA and McA-hAPOB cells were maintained in high-glucose DMEM (Invitrogen, 11965-084) containing penicillin (100 units/mL), streptomycin (100 μg/mL), 10% FBS, and 10% horse serum (Invitrogen). For experiments, all cells were plated on collagen-coated 6-well plates.

### Primary hepatocyte isolation.

Primary hepatocytes were isolated from 8- to 10-week-old male *Sort1*-KO mice or littermate controls fed chow diet ad libitum. Livers were perfused with HBSS without calcium (Invitrogen, 14025-076) and 10 mM HEPES (Invitrogen, 15630-080) through the portal vein (vena cava was severed immediately before perfusion) for 8 minutes (4 mL/min) at a temperature of 37°C. This was followed by a perfusion of high-glucose DMEM with collagenase type I (80 mg/100 mL; Worthington) for 6 minutes (4 mL/min) warmed to 37°C. The liver was removed and transferred to a Petri dish containing 4 mL warm DMEM with collagenase for an additional 2–4 minutes while the tissue was pulled apart with cell scrapers. Cold DMEM (40 mL) was added, and the digested liver was filtered through nylon mesh and collected in a 50 mL conical tube. The cells were centrifuged for 5 minutes at 100*g*. The supernatant was aspirated, and the pellet was washed 3 times with 30 mL ice-cold DMEM. Viable cells were counted after staining with trypan blue. Cells were grown on collagen-coated 6-well plates at a density of 500,000 cells per well in 4 mL DMEM+10% FBS for at least 2 hours. The cells were then washed 2 times with PBS and experiments were begun.

### AAV injection.

8- to 10-week-old C57BL/6J mice were injected i.p. with 3 × 10^11^ GC viral particles of null AAV or mSort1 AAV (12). 8- to 10-week-old *Sort1^fl/fl^* mice were injected with either null AAV or TBG.Cre AAV 1.5 × 10^11^ GC viral particles. All AAV were synthesized by the University of Pennsylvania Vector Core.

### TG and apoB secretion.

An abundance of data in mice and humans indicate that, under certain conditions, the liver can secrete apoB-lipoproteins that are denser and less lipidated than classic VLDL (54, 55). The practical implication of this observation is that a focus solely on *VLDL* apoB secretion can miss apoB that is being secreted in denser particles; hence, we measured total apoB-100 secretion in our studies. Mice that were fasted for 4 hours were injected intravenously with 0.67 mg/g body weight Pluronic F127 NF Prill Poloxamer 407 (P407) (BASF, material 30085239) and 300–400 μCi Easytag EXPRES^35^S protein labeling mix (^35^S Met/Cys) (Perkin Elmer, NEG772). Blood samples were taken immediately prior to the P407/^35^S Met/Cys injection and 30, 60, 90, and 120 minutes after injection. The blood was placed on ice and spun for 7 minutes at 10,000*g*, and plasma was transferred within 15 minutes of collection. Plasma TG concentration was measured at all time points by colorimetric assay (Thermo Fisher Scientific, Infinity TG). TG secretion rate was calculated as the rate of increased plasma TG concentration between 30 and 120 minutes (mg/dl/h).

Newly synthesized and secreted total plasma apoB100 was measured by the incorporation of ^35^S Met/Cys into apoB100 that was secreted into the systemic circulation during the same 2-hour period. Total plasma was boiled in Laemmli sample buffer and separated on a 3%–8% Tris Acetate gel (Novex). The gel was dried onto filter paper under vacuum, and the labeled proteins were visualized by autoradiography. The apoB100 bands were cut and analyzed for [^35^S] activity by liquid scintillation counting. Total plasma was also separated on a 10% Bis Tris gel (Novex), and bands for alb, apoE, and apoA-I were visualized, cut, and counted. To mitigate differences in total labeled hepatic protein synthesis, counts were adjusted based on the incorporation of ^35^S methionine into all proteins in plasma trichloroacetic acid–precipitated (TCA-precipitated) protein counts. To determine TCA-precipitated protein counts, 5 μL plasma from sample was blotted onto a 1 × 1 cm square of filter paper. Plasma proteins were precipitated by incubating the filter paper in 20% TCA on ice followed by 10% TCA heated to 100°C. The filter paper was subsequently rinsed with 100% ethanol and dried, and ^35^S activity was calculated by liquid scintillation counter.

### Transfections.

McA and McA-hAPOB cells were transfected with pcDNA3.1(+) or mSort plasmids using LipofectAMINE 2000 (Invitrogen), according to the manufacturer’s specifications. McA and McA-hAPOB cells were transfected with either ON-TARGET plus nontargeting pool (Dharmacon, D-001810) or ON-TARGET plus rat *Sort1* (83576) siRNA-SMART pool (Dharmacon, L-089536-02). HepG2 cells were transfected with control pool or ON-TARGETplus human *SORT1* (6272) siRNA-SMART pool (Dharmacon, L-010620-00). All siRNA transfections were performed with DharmaFECT4 transfection reagent according to manufacturer’s instructions. Experiments were performed 36–40 hours after transfection.

### Ex vivo and in vitro apoB secretion studies.

Isolated primary hepatocytes, McA cells, and HepG2 cells were incubated with Met/Cys-free DMEM containing 1.5% BSA for 2 hours and then labeled in the same media with 150 μCi/mL ^35^S-Met/Cys for 2 hours. Where noted, the following was added to pretreatment and label media: 0.4 mM, 0.8 mM, or 1.2 mM OA (MilliporeSigma); 0.4 mM PA (MilliporeSigma); 40 μg/mL ALLN (Calpain Inhibitor 1, MilliporeSigma); 10 μM ceramide (Santa Cruz Biotechnology, sc-201375); or 1 μM tunicamycin (MilliporeSigma, T7765). In all experiments, medium was collected and mixed with a protease inhibitor mixture (1 mM benzamidine, 5 mM EDTA, 100 units/mL aprotinin, and 10 mM HEPES, pH 8.0) and 0.86 mM freshly made phenylmethylsulfonyl fluoride. Cells were lysed on ice with lysis buffer containing 62.5 mM sucrose, 0.5% sodium deoxycholate, 0.5% Triton X-100, 50 mM Tris-HCl, pH 7.4, 150 mM NaCl, 50 μg/mL leupeptin, 50 μg/mL pepstatin A, and 30 μL/mL of a protease inhibitor. Cell lysates and media samples were then subjected to immunoprecipitation.

### Pulse-chase experiments.

Other siRNA-transfected McA cells were preincubated in serum-free DMEM without Met/Cys containing 1.5% BSA for 2 hours and then labeled with DMEM containing 1.5% BSA and 200 μCi/mL ^35^S Met/Cys for 20 minutes. After washing, cells were incubated with serum-free DMEM with 1.5% BSA containing excess unlabeled methionine (10 mM) and cysteine (3 mM) for 10 or 120 minutes. Medium was collected at 120 minutes, and cells were lysed at 10 or 120 minutes as described above. Cell lysates and medium were also used for immunoprecipitations.

### Immunoprecipitation.

Immunoprecipitation of proteins was carried out according to the method of Dixon et al. (31). Cell lysate or conditioned medium were mixed with NET buffer (50 mM Tris, pH 7.4, 150 mM NaCl, 5 mM EDTA, 0.5% Triton X-100, 0.1% SDS) and 3 μL anti-apoB antibody (MilliporeSigma, 178467) or anti-alb antibody (MilliporeSigma, A3293). The mixture was incubated at 4°C for 1 hour. Protein A-agarose (Invitrogen) was added to the reaction solution, and the incubation was continued for an additional 16 hours. The beads were washed with NET, and proteins were released with Laemmli sample buffer by boiling for 5 minutes. Samples were separated on 3%–8% Tris Acetate gels, followed by autoradiography or immunoblotting. Media and cell apoB counts from labeling studies were normalized to total protein synthesis, as measured by TCA-precipitable radioactivity in the cell lysate for each sample.

### Sucrose gradient ultracentrifugation.

Sucrose gradient ultracentrifugation of apoB-containing lipoproteins was conducted as described by Boren et al. (63). All solutions contained 0.1 mM leupeptin, 1 μM pepstatin A, 0.86 mM phenylmethylsulfonyl fluoride, 100 units/mL aprotinin, 5 μM ALLN, 5 μM EDTA, 150 mM NaCl, and 50 mM PBS, pH 7.4. The sample layer was prepared by diluting 200 μL pooled plasma from the 120-minute postinjection time point from mice in the apoB secretion studies in 2.3 mL PBS and adding 2.5 mL of 25% sucrose in PBS. The sucrose gradient was formed by layering from the bottom of the tube: 2 mL 47% sucrose, 2 mL 25% sucrose, 5 mL of sample in 12.5% sucrose, and 3 mL PBS. The gradients were spun at 209,000*g* in a Beckman SW41 rotor for 65 hours at 12°C, and then 12 fractions were removed from the top to the bottom and subjected to immunoprecipitation for apoB.

### Gene expression.

Gene expression from liver and cells was measured by quantitative real-time PCR (qRT-PCR). mRNA was isolated and reverse transcribed as previously described. In brief, RNA was extracted from liver and cell samples with Trizol (Thermo Fisher Scientific), and 1 μg RNA was reversed transcribed to cDNA using a High-Capacity cDNA Reverse Transcription Kit (Thermo Fisher Scientific). The cDNA was diluted 40 times and used for qRT-PCR analysis using TaqMan probes for mSort1 (Life Technologies, Mm00490905). All samples analyzed were normalized to the average of 3 housekeeping genes (Actin, Ppia, and Gapdh) using the ΔΔCt method.

### Western blot.

100 mg liver was homogenized in 1 ml lysis buffer containing protease inhibitor (Roche). Cells were collected and protein concentration was measured by BCA protein reagent. Samples were subjected to electrophoresis on appropriate percentage gels and transferred to polyvinylidene-fluoride membranes. Sortilin (Abcam, 16640), apoB (Abcam, 31992), phos eIF2α (Cell Signaling Technologies, 119A11), Grp78 (Stressgen, SPA826), and actin (1:10,000; MilliporeSigma, A5441) were used. All primary antibodies were incubated overnight at 4°C. Species-specific secondary IgG antibodies conjugated with peroxidase (1:10,000 to 1:15,000) were incubated for 1 hour at room temperature. Protein bands were visualized using Immobilion Cresendo or Classico Western HRP Substrate (MilliporeSigma).

### Plasma lipid measurements.

Blood was collected from mice fed either chow or HFD after a 4-hour fast. The blood was spun for 7 minutes at 10,000*g*, and plasma were transferred and frozen. TG and total cholesterol were measured by colorimetric assays (Thermo Fisher Scientific).

### Fast protein liquid chromatography.

Plasma samples collected after a 4-hour fast or 2 hours after Pluronic injection were pooled by experimental group. 150 μL plasma was separated by fast protein liquid chromatography (FPLC) on a Superose TM 6 Increase 10/300 Gl column (GE Healthcare Life Sciences) into fractions each 0.5 mL in volume. Total cholesterol and TG were measured from FPLC-separated fractions using Infinity cholesterol and TG reagents (Thermo Fisher Scientific) in 96-well microplates with a Synergy Multi-Mode Microplate Reader (BioTek).

### UK biobank.

The UK biobank is a population-based cohort study conducted in the United Kingdom from 2006 to 2010; 502,505 volunteers, aged 37–73 years at baseline, were recruited. All participants were registered with the UK National Health Service and were encouraged to go to an assessment center for an initial examination, which was followed by a long-term follow-up through various health data systems. Our study population comprises the baseline assessment (2006–2010), in which the participants provided blood samples and clinical data (e.g., presence of diabetes mellitus) as well as physical measures (e.g., BMI). All participants gave informed consent for genotyping and data linkage to medical reports. Genotyping of the *SORT1* variant rs12740374_T was conducted in a total of 487,427 White participants.

TGs were measured by GPO-POD analysis on a Beckman Coulter AU5800 and ApoB was measured by immunoturbidimetric analysis on a Beckman Coulter AU5800.

In a subset of the selected UK biobank patients metabolomic analyses were performed (*n =* 103,467). LDL was measured by enzymatic protective selection analysis on a Beckman Coulter AU5800. Concentration of VLDL was measured by Nightingale Health using high-throughput NMR-based metabolic biomarker profiling.

All continuous variables were analyzed by unpaired, 2-tailed *t* tests for univariate analysis as well as by a multivariable linear model to account for relevant confounders. All categorical variables were displayed as relative (%) frequencies, and the corresponding contingency tables were analyzed using the χ^2^ test. Multivariable logistic regression was performed to test for independent associations. All multivariable analyses were adjusted for age, sex, BMI, and presence of diabetes mellitus. To compare differences between *SORT1* rs12740374_T heterozygotes and noncarriers, relative differences between *SORT1* rs12740374_T carriers and noncarriers were calculated. Each group was compared with the rs12740374_T noncarriers using a multivariable linear model to account for cofactors. Here, adjustment for age, sex, and BMI were performed. Differences were considered to be statistically significant when *P <* 0.05. The data were analyzed using R version 4.0.2 (R Foundation for Statistical Computing), SPSS Statistics version 26 (IBM), and Prism version 8 (GraphPad).

### Statistics.

Statistical details of experiments are described in the figure legends. Data are presented as mean ± SD unless otherwise stated. Differences in the mean values between 2 groups were assessed using 2-tailed Student’s *t* test. Differences in mean values among more than 2 groups were assessed by 1-way ANOVA, with any individual group differences determined using Tukey’s test. *P* < 0.05 was considered to be statistically significant. TG secretion was analyzed by repeated-measures 2-way ANOVA.

### Study approval.

All procedures were approved by the University of Pennsylvania Institutional Animal Care and Use Committee. This study was approved by the UK biobank under the application 70653.

## Author contributions

DMC and DJR conceptualized the study. DMC provided methodology. DMC, CVS, and YAK provided formal analysis. DMC, CVS, AR, and KG provided investigation. DMC and DJR wrote the original draft. DMC, CVS, NJH, and DJR reviewed and edited the manuscript. DMC and DJR acquired funding. NJH and DJR supervised the study.

## Supplementary Material

Supplemental data

## Figures and Tables

**Table 1:**
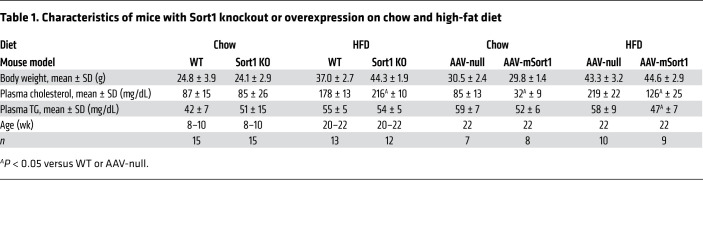
Characteristics of mice with Sort1 knockout or overexpression on chow and high-fat diet

## Version 1 02/03/2022

In-Press Preview

## Version 2 03/15/2022

Electronic publication
