# Supplementary material for: Sortilin restricts secretion of apolipoprotein B-100 by hepatocytes under stressed but not basal conditions
Source: J Clin Invest. 2022 Mar 15;132(6):e144334. doi: 10.1172/JCI144334 (PMC8920325; doi:10.1172/JCI144334)
Supplement: Supplemental data [file jci-132-144334-s117.pdf]

Supplemental Data

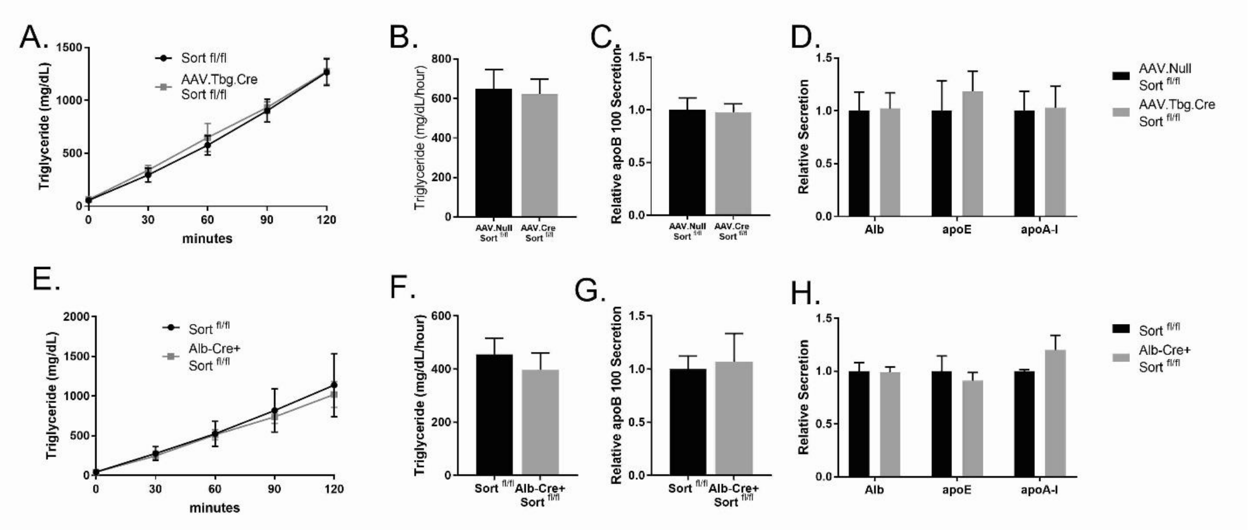

Figure S1- A) 8-10 week old male Sort1$^{fl/fl}$ were injected with AAV.Tbg.Cre or null AAV and after 3 weeks mice were injected IV with pluronic and ^{35}S Met/Cys and bled every 30 minutes for 2hours. Plasma TG was measured B)TG secretion rate was calculated as the mg/dL/hour. C) Total plasma from each mouse from the 2 hour post-injection time point was subjected to autoradiograpy and ^{35}S labeled apoB100 and D) ^{35}S labeled Alb, apoE, and apoA-I bands was counted. N=8/group E) 8-10 week old male Sort1$^{fl/fl}$ and AlbCre+Sort1$^{fl/fl}$ (LSKO) mice were injected IV with pluronic and ^{35}S Met/Cys and bled every 30 minutes for 2hours. Plasma TG was measured F)TG secretion rate was calculated as the mg/dL/hour. G) Total plasma from each mouse from the 2 hour post-injection time point was subjected to autoradiograpy and ^{35}S labeled apoB100 and H) ^{35}S labeled Alb, apoE, and apoA-I bands was counted. N=8/group

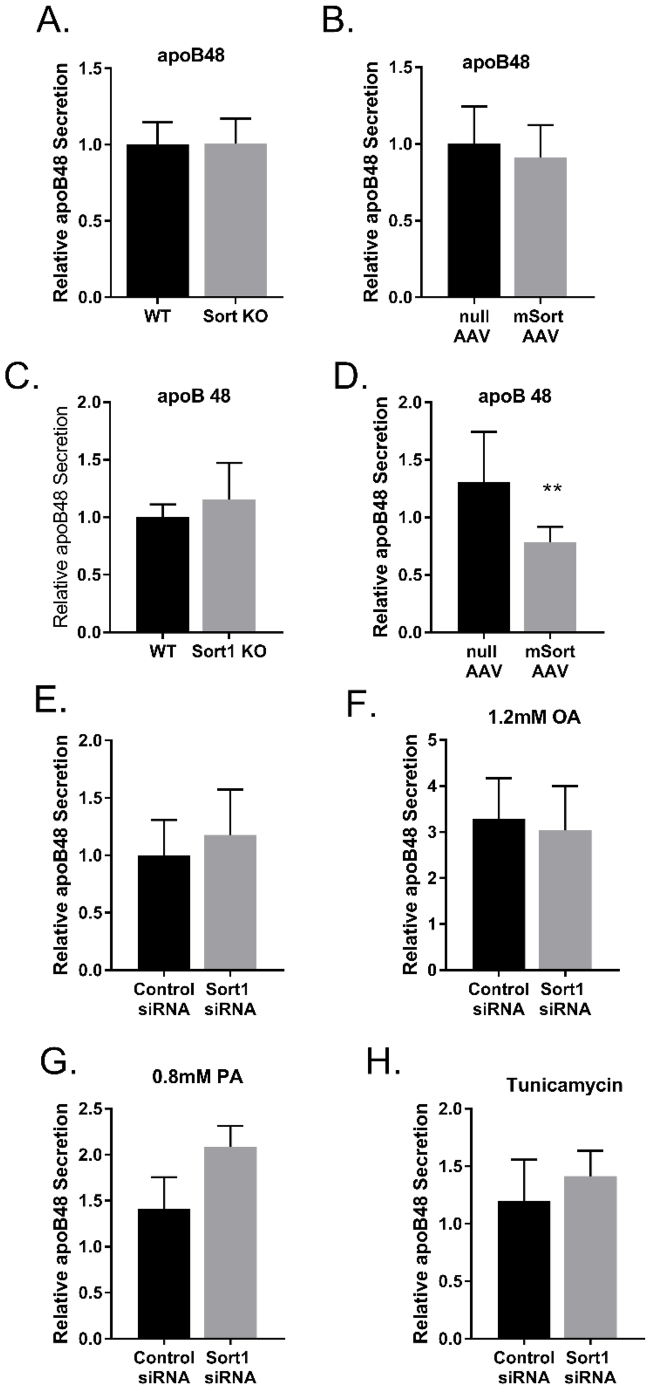

Figure S2 –Newly synthesized apoB48 secreted in A) chow fed Sort1 KO mice and littermate controls B) chow fed C57BL6/J mice treated with Sort1 or null AAV C) High fat diet fed Sort1 KO mice and littermate controls D) High fat diet fed C57BL6/J mice treated with Sort1 or null AAV E) McA cells with Sort1 knockdown F-H) McA cells with Sort1 knockdown treated with 1.2mM oleic acid (F), 0.8mM PA (G), or 1uM Tunicamycin (H)

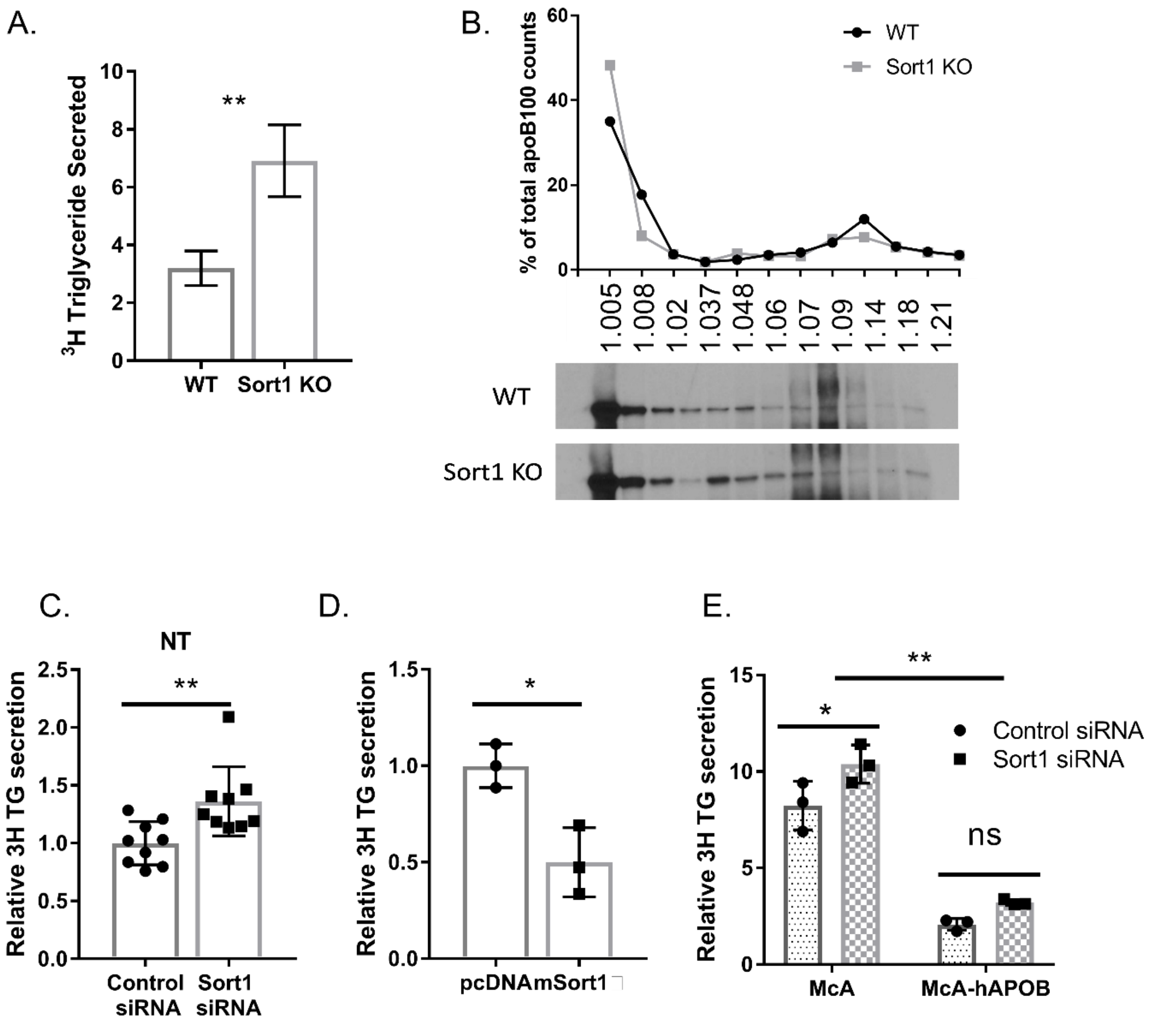

Figure S3- A) Isolated primary hepatocytes from wildtype or Sort1 knockout mice were labeled with with 10uCi of 3H glycerol (American Radiolabeld Chemicals) for 4 hours in DMEM + 1.5% BSA The media was collected and subjected to Folch extraction and separated by Thin Layer Chromatography. Separate wells were analyzed for ^{3}H activity in TG spot and normalized to total cell protein as measured by BCA assay. *P< 0.05 by student's t test. N=3 wells per mouse from 3 mice/group. B) Isolated primary hepatocytes in a 10cm dish were labeled with 35S met/cys for 2 hours and the media was collected and separated by density using a sucrose density ultracentrifugation spin. Fractions were then immunoprecipitated with anti-apoB antibody and visualized by autoradiography. C-E) McA-RH7777 (McA) and McA-HAPOB cells were transfected with either Sort1 siRNA or m*Sort1* or empty control vector (pcDNA) vector and labeled with 3H glycerol and 3H TG measured as described above. *P< 0.05 by student's t test in panels C and D. *P≤0.05 by 1 way ANOVA in panel E, N=3-9 per cell line,

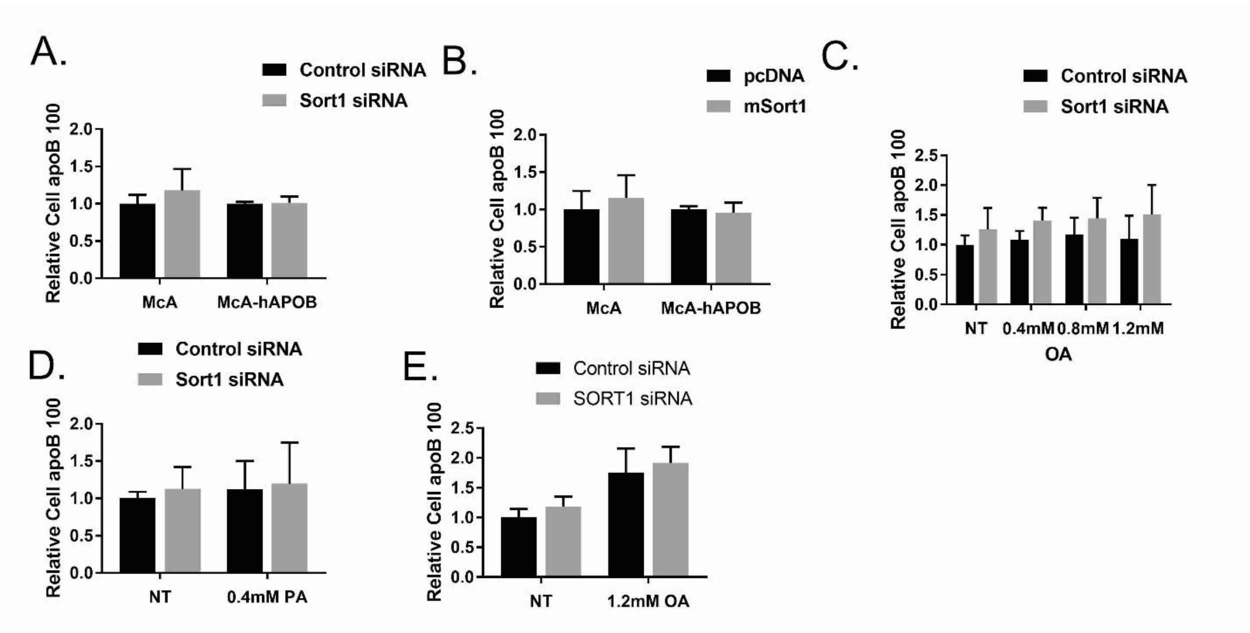

Figure S4- McA-RH7777 (McA) and McA-HAPOB cells were transfected with rSort1 siRNA or control siRNA. After 36 hrs, cells were incubated for 1 hour in DMEM without Met/Cys+1.5% BSA and then labeled in the same media with ^{35}S Met/Cys for 2 hours. Media was collected and cells were lysed and subjected to autoradiography and ^{35}S labeled apoB100 were counted and normalized to total ^{35}S trichloroacetic acid precipitible counts. N=3 wells/group Representative of 3 independent experiments B) McA and McA-HAPOB cells were transfected with mSort1 or control vector (pcDNA) and cell apoB100 was measured as described in panel A N=3 wells/group Representative of 3 independent experiments C) siRNA transfected McA cells were incubated in DMEM without Met/Cys+1.5% BSA with 0(NT), 0.4, 0.8, or 1.2mM Oleic Acid (OA) for 2 hours and then labeled in the same media with ^{35}S Met/Cys for 2 hours after which cell apoB100 was measured n=9wells/ treatment/ group. D) cell apoB100 from McA cells treated with 0 or 0.4mM Palmitic acid (PA), n=9 well/treatment/group E) cell apoB100 secretion in siRNA transfected HepG2 cells treated with 0 or 1.2mM OA, n=9 well/treatment/group . *$p<0.05$ vs control siRNA or pcDNA by ANOVA.

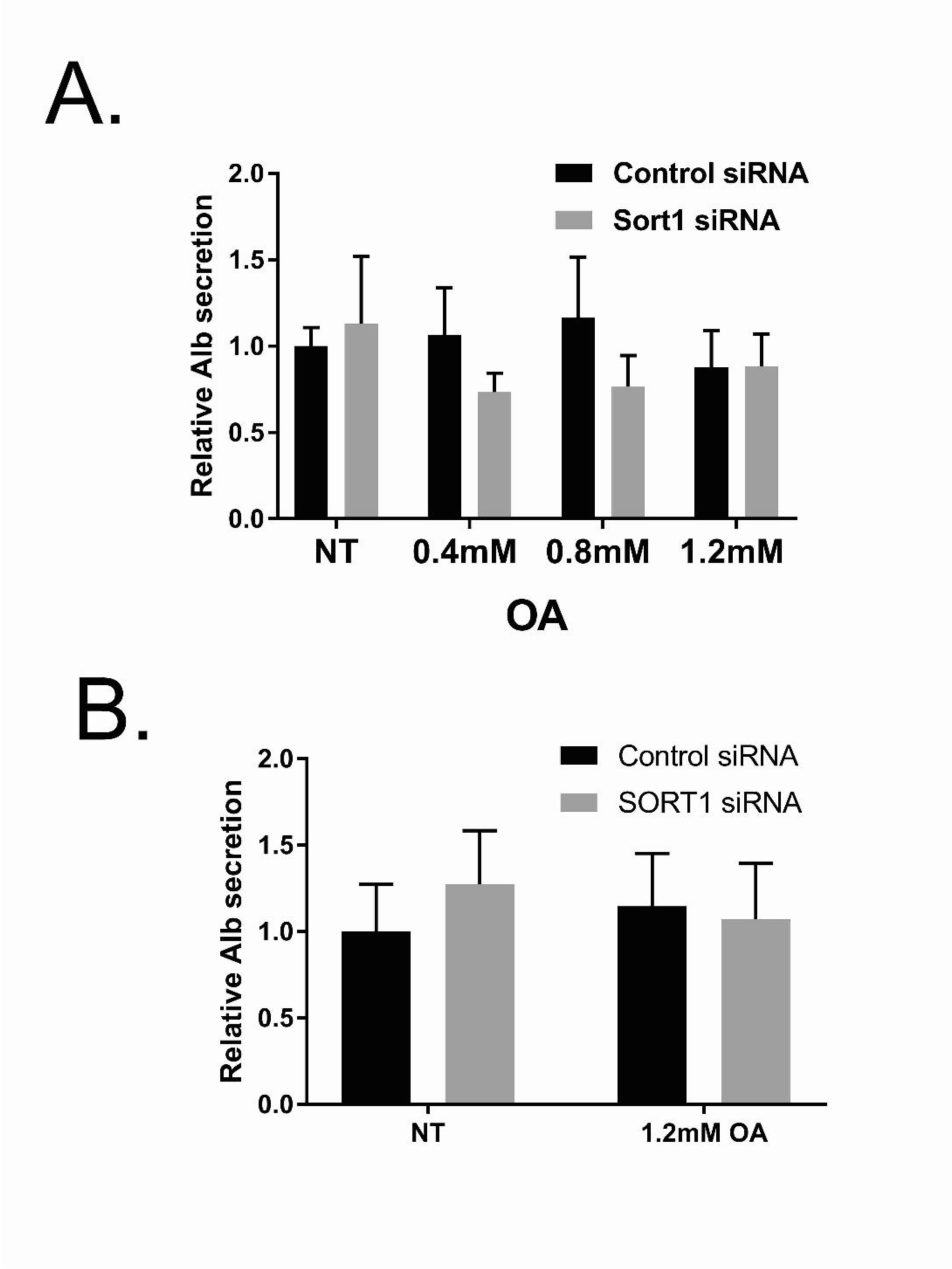

Figure S5- A) McA-RH7777 (McA) cells were transfected with rSort1 siRNA or control siRNA. After 36 hrs, cells were incubated in DMEM without Met/Cys+1.5% BSA with 0(NT), 0.4, 0.8, or 1.2mM Oleic Acid (OA) for 2 hours and then labeled in the same media with ^{35}S Met/Cys for 2 hours. Media was collected and cells were lysed and subjected to autoradiography and ^{35}S labeled alb were counted and normalized to total ^{35}S trichloroacetic acid precipitible counts. N=6 wells/group. B) Alb secretion from HepG2 cells treated with 0 or 1.2mM OA, n=6 well/treatment/group

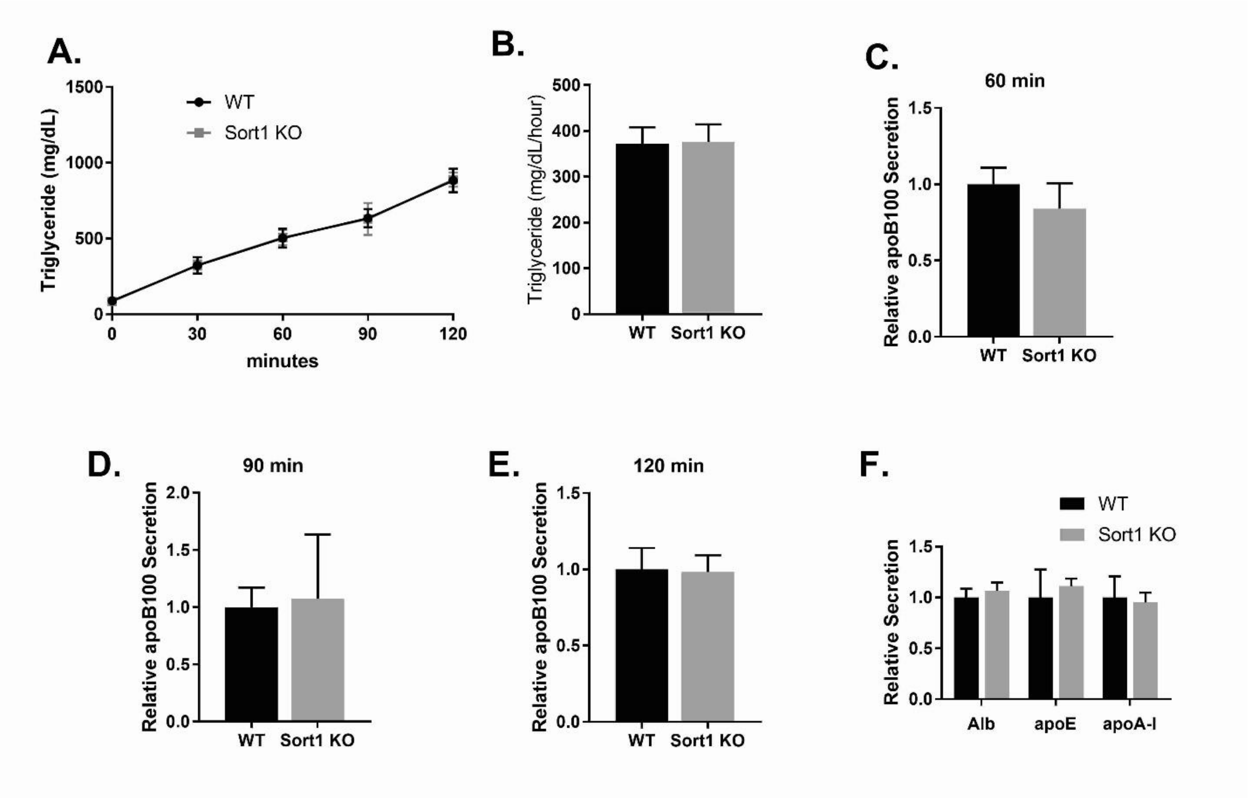

Figure S6- A) 22 week old Sort1 KO mice fed a chow diet were injected IV with pluronic and ^{35}S Met/Cys and bled every 30 minutes for 2 hours. Plasma TG was measured B)TG secretion rate was calculated as the mg/dL/hour. C-E) Total plasma from each mouse at the post-injection time point was subjected to autoradiograpy and ^{35}S labeled apoB100 and F) ^{35}S labeled Alb, apoE, and apoA-I bands was counted. N=8/group from 2 experiments

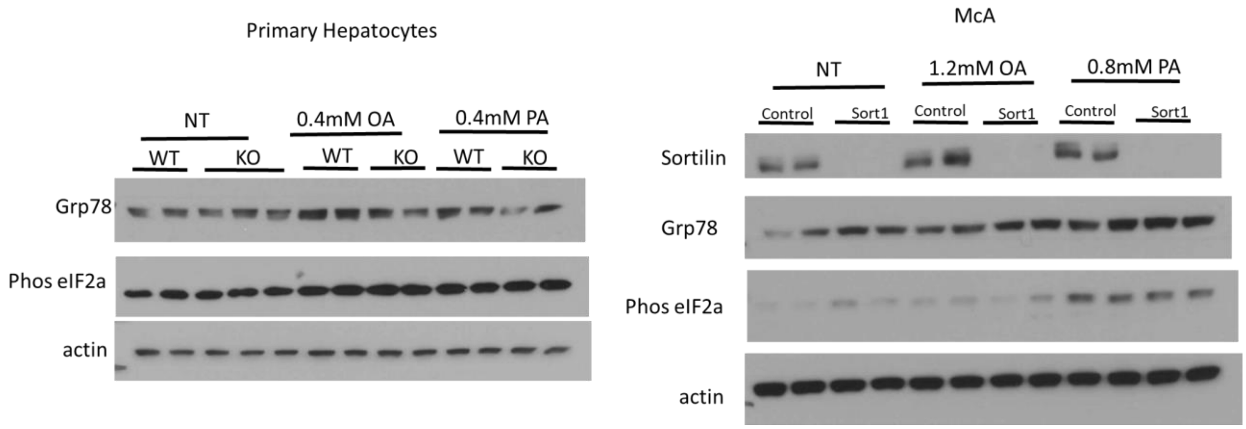

Figure S7 –A) Isolated mouse primary hepatocytes were treated with 0.4mM OA or 0.4mM PA for 4 hours and thenwere lysed and western blotted for Grp78, phos-eIF2a and actin protein. B) siRNA treated McA cells were incubated with 1.2mM OA or 0.8mM PA for 4 hours and then were lysed and western blotted for Sortilin, Grp78, phos-eIF2a and actin protein.

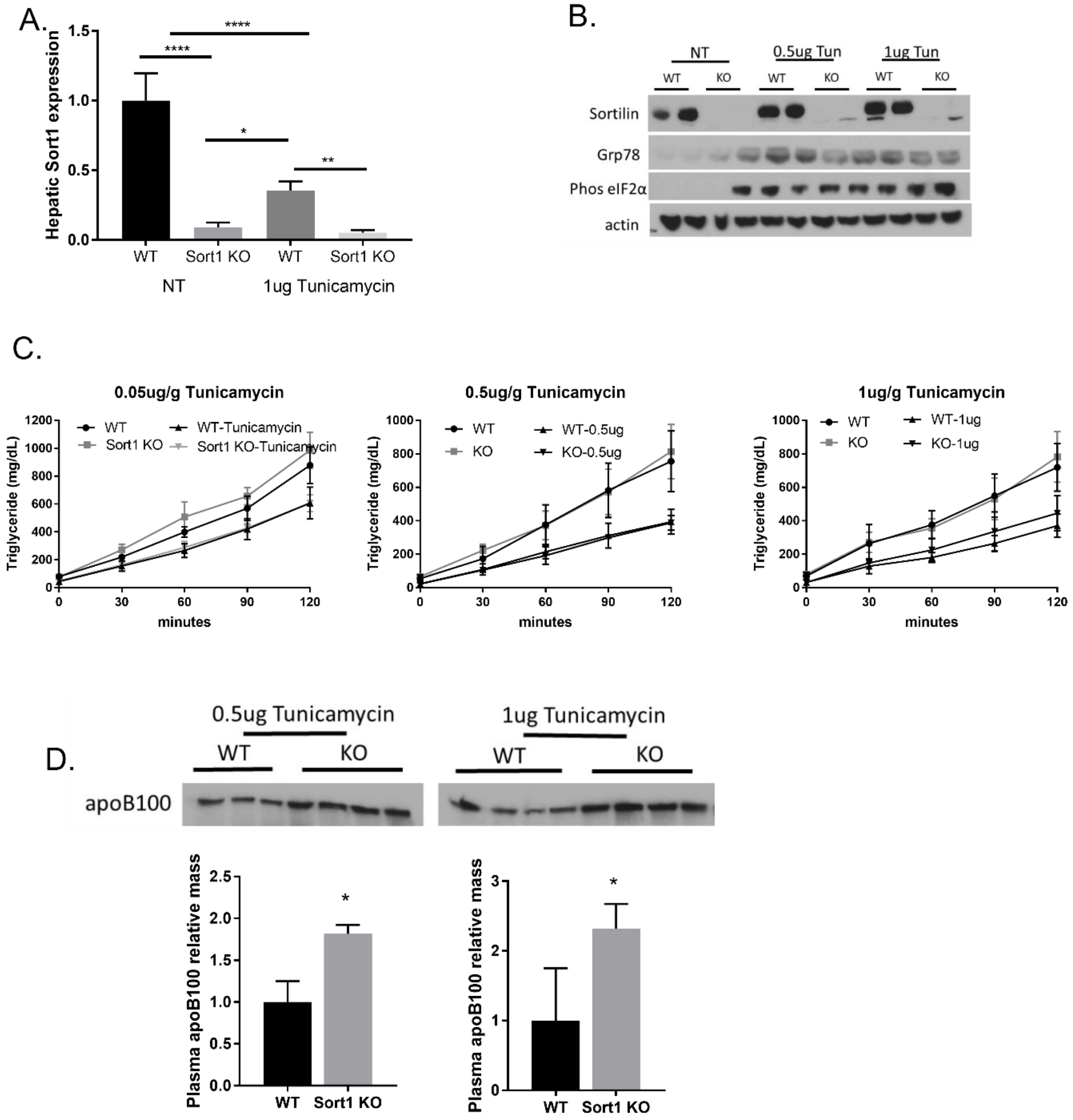

Figure S8 - WT and Sort1 KO mice were injected IP with 0.05, 0.5 or 1ug/g body weight Tunicamycin. After 4 hours, mice were injected with pluronic and bled after 2 hours. A) Liver Sort1 expression was measured, * $p<0.05$ by ANOVA B) Livers were homogenized and western blotted for Sortilin, Grp78, phos-eIF2a and actin protein. C) Plasma TG was measured D. 2 hour post-pluronic plasma was immunoblotted for apoB and quantified by densitometry. *$p<0.05$

Supplemental Table 1

Characterization of 8-12 week old Hepatocyte-Specifc *Sort1* Knockout Mice

	**chow**		**chow**	
	**Flox**	**Alb Cre**	**Flox**	**AAV Cre**
**Body Weight**	**27.04**	**24.84**	**32.15**	**31.32**
**(g)**	2.01	2.95	5.1	4.23
**plasma Cholesterol**	**73.48**	**87.5**	**90.33**	**91**
**(mg/dL)**	17.9	17.06	14.01	14.62
**Plasma TG**	**47.15**	**39.86**	**55.2**	**62.96**
**(mg/dL)**	3.96	18.19	13.63	20.5
**n**	11	13	12	12
